# On-Device Smartphone Vibration Analysis for Passenger-Car Ride Comfort Evaluation

**DOI:** 10.3390/s26206592

**Published:** 2026-10-17

**Authors:** Mahalingam Nainaragaram Ramasamy, Jiří Tůma, Narendren Senthil, Aleš Slíva, Jan Dizo

**Affiliations:** 1 Faculty of Mechanical Engineering, VSB Technical University of Ostrava, 17. Listopadu, 15/2172, 70 800 Ostrava, Czech Republicnarendren.senthil.st@vsb.cz (N.S.); ales.sliva@vsb.cz (A.S.); 2 Faculty of Mechanical Engineering, University of Zilina, 17. Univerzitna 8215/1, 010 26 Zilina, Slovakia; jan.dizo@fstroj.uniza.sk

**Keywords:** ride comfort, smartphone accelerometer, ISO 2631-1, passenger cars, vibration measurement, power spectral density

## Abstract

**Highlights:**

Smartphone app with an on-device ISO 2631-1-aligned processing pipeline ranked ride comfort across three vehicles/two speeds− Passat best at 50 km/h (a_w_ = 0.272 m·s^−2^, 97%), Astra worst at 30 km/h (a_w_ = 0.812, “uncomfortable”). Comfort differences traced to sub-8 Hz sprung-mass content, while distinct 25–50 Hz seat resonances shaped harshness without driving the score.

**What are the main findings?**

- Comfort ranking based on weighted RMS acceleration: Octavia > Passat > Astra at 30 km/h, whereas Passat > Octavia > Astra at 50 km/h.
- Two resonance families: body bounce (~1–1.4 Hz)/wheel hop (~11–13 Hz), plus vehicle-specific seat modes (25–50 Hz).

**What are the implications of the main findings?**

- Repeatability confirmed: mean aw = 0.170 m·s^−2^, CoV = 13% across five runs.

**Abstract:**

This study presents a smartphone-based approach for passenger-car ride comfort evaluation using an Android application that performs vibration acquisition and signal processing directly on the device. The Ride Comfort application records tri-axial acceleration and computes FFT, Welch power spectral density (PSD) with a 66.66% overlapping Hann window, 1/3-octave spectra, and frequency-weighted RMS acceleration following ISO 2631-1 principles. Six comparative field tests were conducted with an Opel Astra, Skoda Octavia, and Volkswagen Passat at approximately 30 km/h on Track A and 50 km/h on Track B. At 30 km/h, the Octavia, Passat, and Astra recorded a_w_ values of 0.668, 0.712, and 0.812 m·s^−2^, respectively. At 50 km/h, the Passat, Octavia, and Astra recorded 0.272, 0.432, and 0.539 m·s^−2^, respectively. Five repeatability tests with a Skoda Fabia at approximately 90 km/h on a fixed 3 km highway segment yielded a mean a_w_ of 0.169 m·s^−2^, SD of 0.022 m·s^−2^, and CoV of 13.0%. The results demonstrate the feasibility of an integrated smartphone workflow for ride comfort assessment and repeatable measurement under a fixed vehicle–road–speed condition.

## 1. Introduction

Ride comfort is a crucial aspect of vehicle performance, impacting passenger experience, safety, and long-term health [1]. Measurement of comfort level relies on the ISO 2631-1 standard, which defines how to compute frequency-weighted RMS acceleration values to align with human perception and categorizes comfort level into multiple zones ranging from “not uncomfortable” to “extremely uncomfortable” [2].

**Figure 1:**
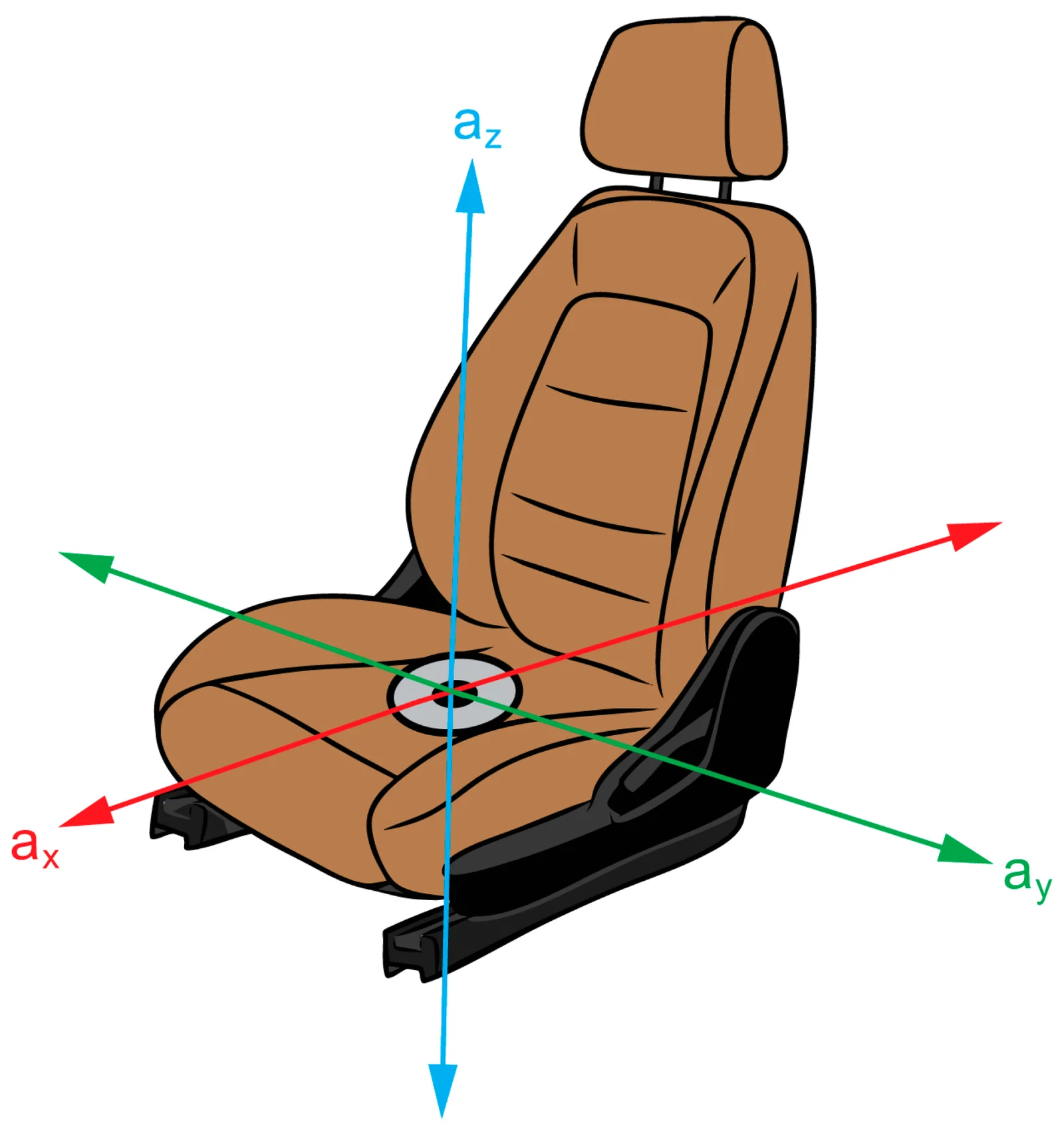
Human body vibration axes (a_x_, a_y_, a_z_) and sensor position on the vehicle seat.

Along with ISO 2631-1, the SAE J1490 standard offers a proper guideline for evaluating ride comfort in road vehicles. SAE J1490 provides the required procedure for measuring whole-body vibration in seated passengers using tri-axial accelerometers. Additionally, recommendations are provided for the placement and position of the sensor, including its alignment, mounting configurations, and vibration axis orientation, as shown in Figure 1, which are highly significant to driver and passenger postures [3]. This standard also emphasizes the use of frequency-weighting filters to approximate the human biomechanical response across various frequencies [4].

Several studies have been implemented alongside ISO 2631-1 to compare vibration intensities across different road types and vehicle categories [5,6]. Signal processing methods such as Fast Fourier Transform (FFT) and Power Spectral Density (PSD) estimation are central to analyzing vibration data for comfort assessment. They allow researchers to decompose complex motion into frequency components, enabling comparison with ISO-defined sensitivity curves. For example, frequency domain analysis has been applied in road–vehicle studies to evaluate ride-related vibration and road-surface roughness [5,7]. A 1/3rd-octave band analysis, another method aligned with ISO 2631-1, enables mapping the vibration power distribution across standardized frequency bands, aiding in the identification of resonance-related discomfort [8]. Traditional ride comfort studies employ laboratory-grade accelerometers, data acquisition systems (DAQs), and signal analyzers to capture and process vibration data. Such setups, while precise, are often cost prohibitive and logistically limited in scalability for large-scale or on-road studies [9]. Recent advances in smartphone hardware have transformed this landscape with embedded inertial measurement units (IMUs). Particularly, accelerometers can now offer sufficient sensitivity to capture the whole-body vibration required for the ride comfort analysis.

On roads, studies have estimated roughness and ride quality with vehicle-mounted phones, relating acceleration features to the pavement condition and demonstrating city-scale potential, often with off-device post-processing and heterogeneous mounting [10,11]. Cargnin, Lunkes, and de Souza compared a smartphone accelerometer against a dedicated human vibration analyzer on a medium sedan, computing ISO 2631-1 RMS; the smartphone stood within ~5% variation [12]. A recent measurement framework validated a phone IMU on a shaking table and then on-road, reporting >90% consistency with ISO 2631-1 comfort classifications suitable for urban-scale monitoring [13]. Complementing these app-based directions, Silva et al. described the design of a dedicated whole-body vibration device, reinforcing best practices for calibration and repeatability that smartphone studies can emulate [14]. Rana and Asaduzzaman also proposed a low-cost pavement roughness monitoring approach that uses a smartphone’s accelerometer with vehicle dynamics modeling and estimated vehicle parameters to reconstruct road roughness [15]. Recent passenger-car studies have also emphasized the importance of whole-body vibration measured at the seat–occupant interface and the influence of seat–human coupling on perceived ride comfort [16,17]. These findings further support the use of seat-level vibration measurements and ISO 2631-1-based interpretation in passenger-car comfort assessment.

Previous research includes smartphone and conventional vibration assessment systems with different objectives, signal processing procedures, and levels of on-device computation. Table 1 summarizes these methodological distinctions.

Table 1 distinguishes existing approaches by their objectives, analysis methods, and processing architectures. The contribution of the present work therefore lies in integrating the complete acquisition, processing, and interpretation workflow within a single smartphone application, rather than in the use of smartphone accelerometers itself. The resulting platform provides a portable and low-cost approach for ISO 2631-1-aligned ride comfort assessment without requiring external computational resources.

## 2. Materials and Methods

**Figure 2:**
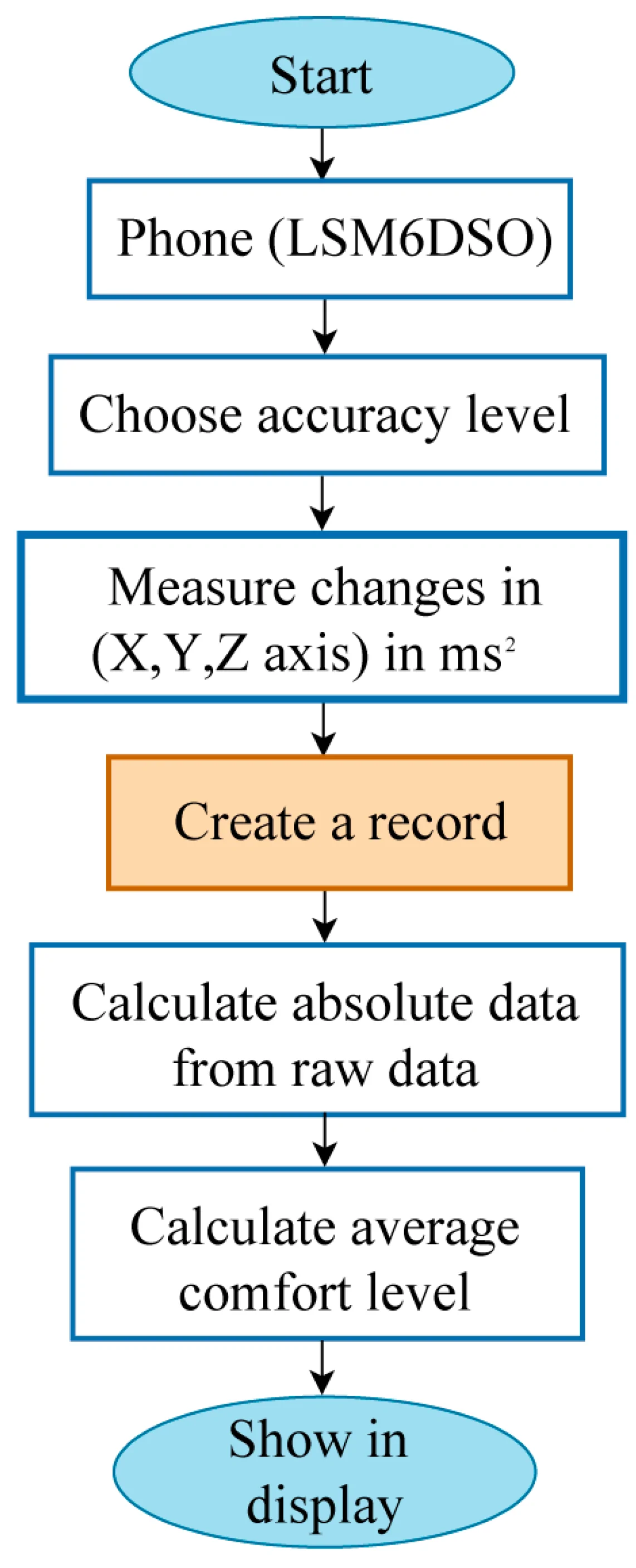
Workflow of the Ride Comfort Android application for vibration data acquisition, signal processing, and ride comfort evaluation.

This study adopted an experimental methodology to evaluate ride comfort using smartphone-based vibration measurements collected under real-world driving conditions. A custom-developed Android application, “Ride Comfort”, was used to acquire and process tri-axial acceleration data from the smartphone’s internal sensors. The flowchart shown in Figure 2 explains the basic workflow of the Android application.

This methodology has four main components: comfort evaluation criterion, test vehicles and their environment, smartphone hardware, and mobile application design for real-time signal acquisition.

### 2.1. Comfort Evaluation Criterion

ISO 2631-1 is an internationally accepted standard for assessing the impact of body vibrations on human comfort and well-being. It outlines how to record the acceleration experienced by people sitting, standing, or lying down [2,21].

The vertical vibration is evaluated using a weight filter, highlighting the RMS (aw) values that are most sensitive to discomfort [22].

(1) $$a_{wf}=\sqrt{\sum_{k=k_{min}}^{k_{max}}W_{k}^{\;2}\;P\left(k\right)}$$

where RMS $a_{wf}$ is frequency-weighted RMS acceleration, m/s^2^; *P*(*k*) is acceleration power associated with frequency bin k; W_k_ is the ISO 2631-1 frequency weighting factor at frequency bin k; and k is the discrete frequency bin index used in the summation when processing the FFT [22]. Table 2 shows various levels of vibration exposure in terms of acceleration that are acceptable for the human body and its comfort level according to the ISO 2631-1 standard.

In addition to the ISO 2631-1 comfort levels, the Ride Comfort application displays an extended percentage-based scale (0–100%) for ease of interpretation. The percentage-based scale can be expressed as,

(2) $$Pa_{wf}\left(\%\right)=\left\{\begin{array}{c} 100,\;\;a_{wf}\leq \;0\\ 97+\left(100-97\right)\frac{0-a_{wf}}{0-b_{0}},\;\;0<a_{wf}<b_{0}\\ y_{i-1}\;\left(y_{i}-y_{i-1}\right)\frac{a_{wf}-b_{i-1}}{b_{i}-b_{i-1}},{\;b}_{i-1}\leq \;a_{wf}\;\leq \;{\;b}_{i};\left(For\;i=1,\ldots ,7\right)\\ \max(0,\;11-{(a}_{wf}-b_{8})\cdot k),\;\;a_{wf}>b_{8};(k=10\%/m\cdot s^{-2}) \end{array}\right.$$

where (*a_wf_*) is the measured frequency-weighted RMS acceleration, (*b_i_*) and (*b*_*i*+1_) are the lower and upper acceleration breakpoints of the corresponding interval, and (*y_i_*) and (*y*_*i*+1_) are the associated lower and upper percentage-scale value. The breakpoint values b_i_ were not selected empirically in the present experiments. They were derived from the boundary values of the ISO 2631-1 comfort categories listed in Table 2. Specifically, the breakpoints were (*b*_0_ = 0.32), (*b*_1_ = 0.50), (*b*_2_ = 0.63), (*b*_3_ = 0.80), (*b*_4_ = 1.00), (*b*_5_ = 1.25), (*b*_6_ = 1.60), (*b*_7_ = 2.00), and (*b*_8_ = 2.50) m·s^−2^. The first value, 0.32 m·s^−2^, represents the rounded value of the ISO boundary of 0.315 m·s^−2^. These values correspond to the limits of the overlapping qualitative comfort ranges defined in ISO 2631-1. The percentage scale itself is not defined by ISO 2631-1; it is an application-specific visualization obtained by interpolation between these ISO-derived breakpoints to provide users with a more granular display. Therefore, the percentage value should be interpreted only as a supplementary display index, while the frequency-weighted RMS acceleration a_w_ and its ISO comfort category remain the primary assessment quantities.

**Figure 3:**
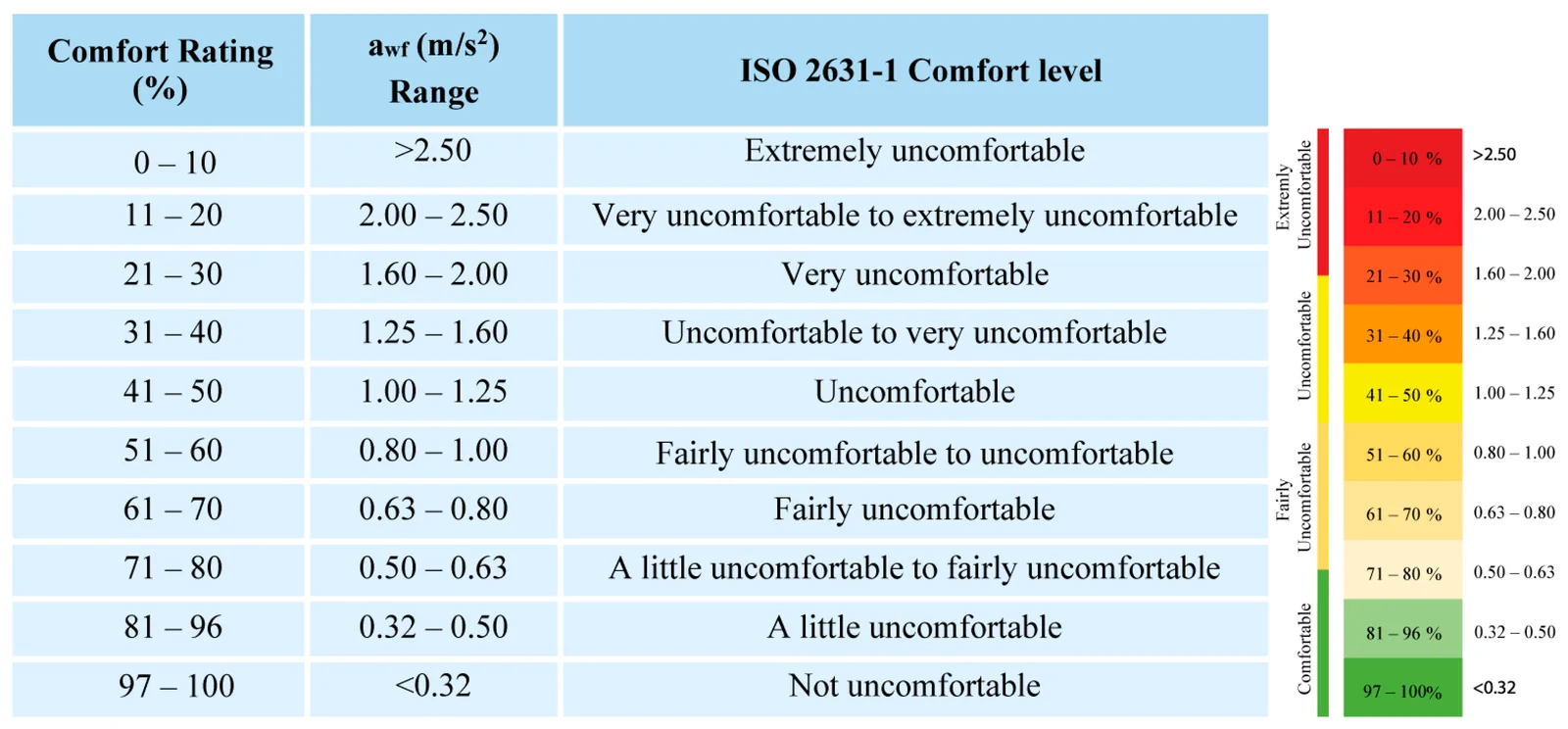
Comfort rating scale to interpret results.

This percentage does not come directly from ISO 2631-1 but was implemented as an internal mapping in the application to provide more granular feedback for the user, as shown in Figure 3.

This application’s algorithm maps each measured RMS value to its corresponding comfort rating and color code, which is then displayed to the user alongside raw and processed vibration data. This integration allows the app to present both the quantitative RMS metric and its qualitative comfort interpretation, which enables users to understand the results correctly even without knowledge of vibration analysis.

### 2.2. Test Vehicles and Environments

**Figure 4:**
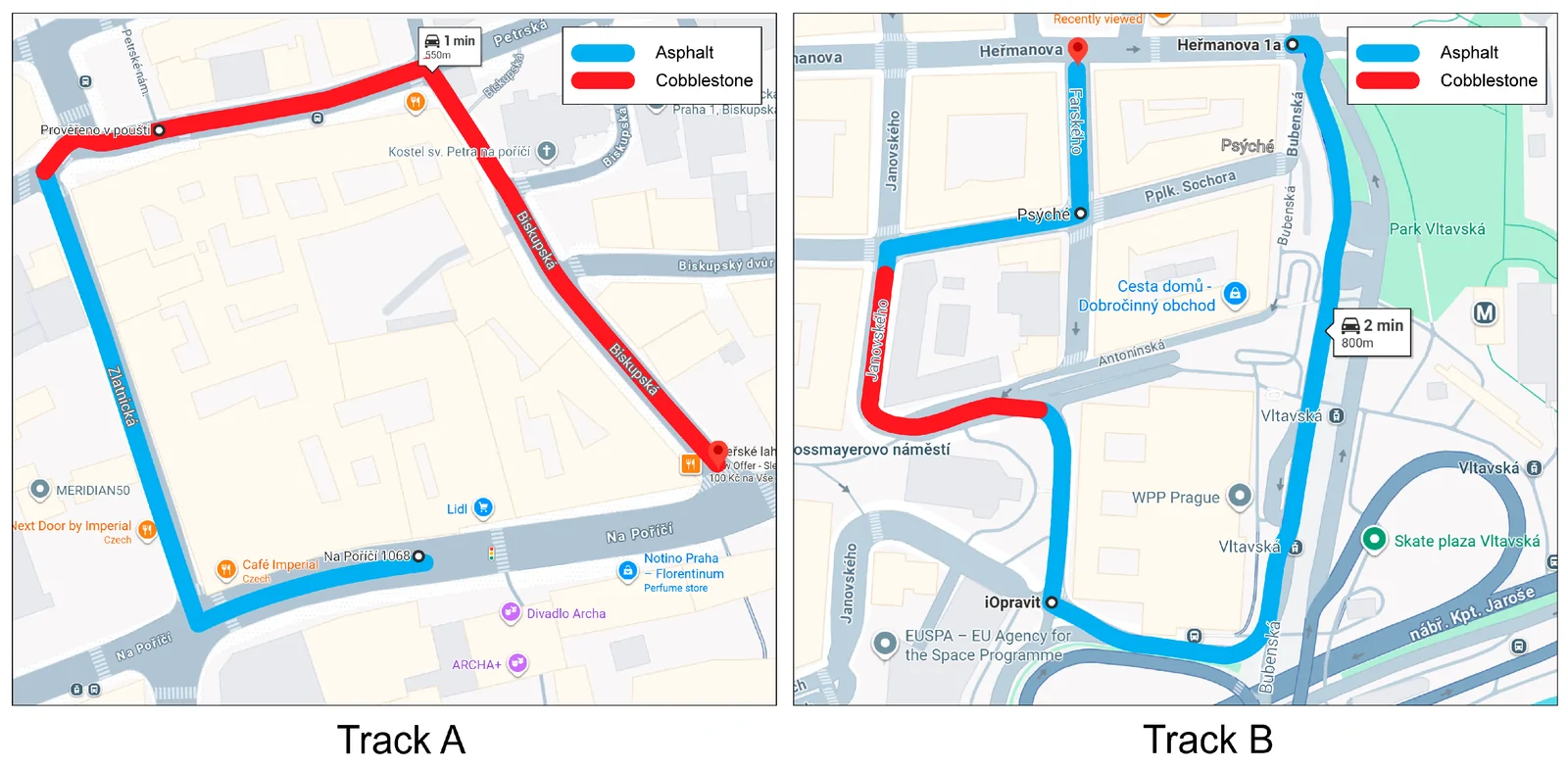
Track A used for testing cars at 30 km/h, and Track B used for testing cars at 50 km/h.

Field measurements were conducted using three different vehicles at average speeds of 30 km/h and 50 km/h in harsh multi-terrain road conditions in the city of Prague. The vehicle speed was not constant during the testing; the study reported the approximate speed. The test has been carried out on two different tracks, A and B, as shown in Figure 4. Track A has approximately the first 220 m of asphalt, followed by 330 m of cobblestones, and Track B has approximately 160 m of cobblestones and 640 m of asphalt. The two speed conditions were not crossed between the two tracks; therefore, the 30 km/h and 50 km/h measurements represent two combined road–speed test conditions, and differences between them should not be interpreted as effects of vehicle speed alone. Table 3 shows the vehicle model, test number, approximate speed, test duration, and test distance. The market share clearly shows that ~35.4% of cars belong to segment C and ~14.9% to segment D. Vehicles were chosen based on their segment and market share [23,24].

### 2.3. Repeatability Test Protocol

**Figure 5:**
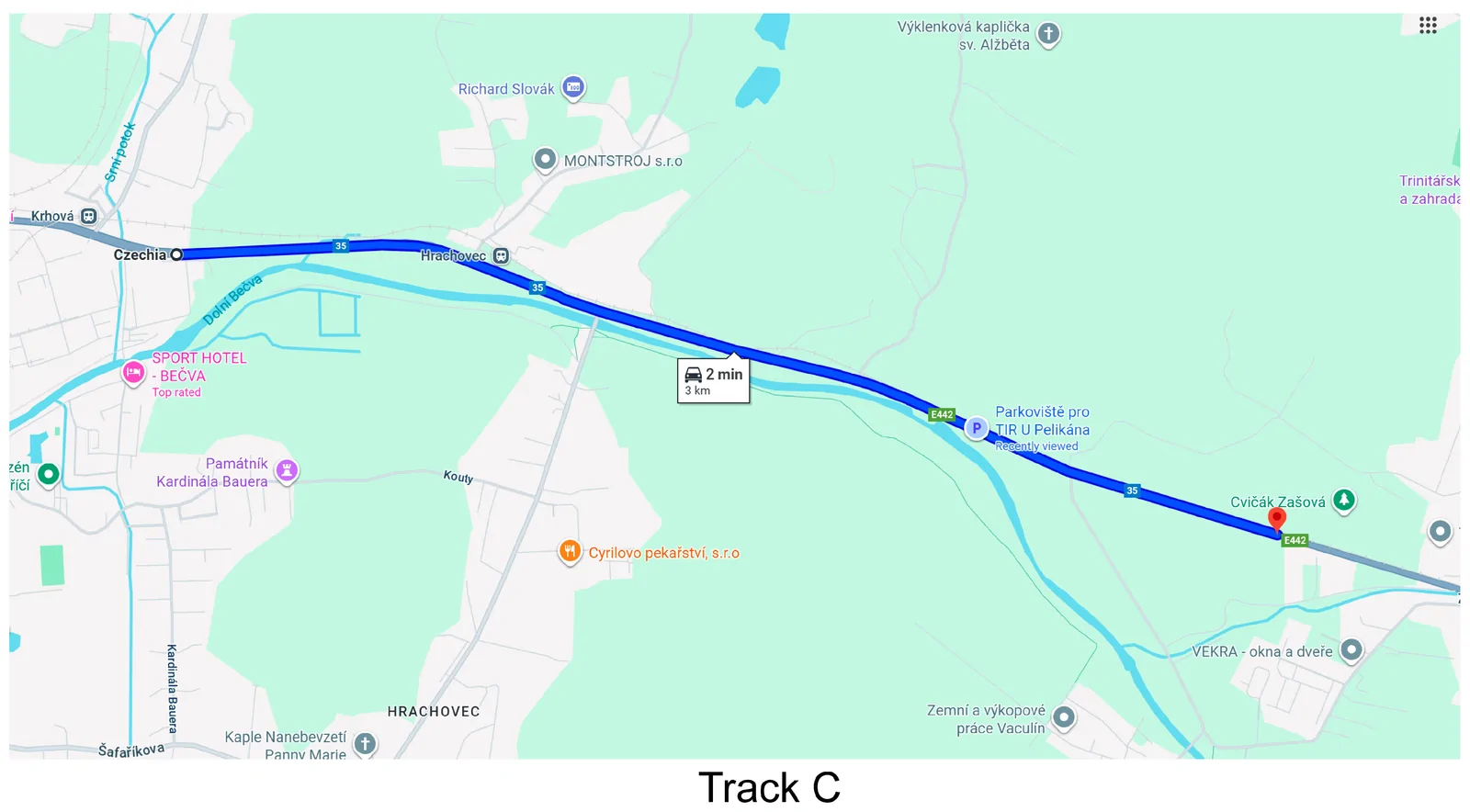
Track C (E442 Highway) used for testing at a speed of 90 km/h.

To verify the application’s measurement reliability, a repeatability test was conducted 5 times using a Skoda Fabia on Highway E442 at 90 km/h, covering 3000 m, for approximately 2 min, as shown in Figure 5. The distance, air and track temperature, wind speed and direction, relative humidity, tire pressure and tread depth on all four wheels, and gear engagement were recorded for each test according to ISO 15037-3:2022 (refer to Table 4 and Table 5) [25]. The repeatability was summarized in Section 4 and Section 5 by the mean, standard deviation (SD), coefficient of variation (COV), and 95% confidence intervals across the five runs.

### 2.4. Smartphone Hardware

The test used a commercially available Android smartphone equipped with an LSM6DSO Micro-electromechanical Systems (MEMS) accelerometer with specifications of 16-bit resolution, measuring ranges from ±2 g to ±16 g, and a noise sensitivity of 70 to 110 μg/√Hz [26].

**Figure 6:**
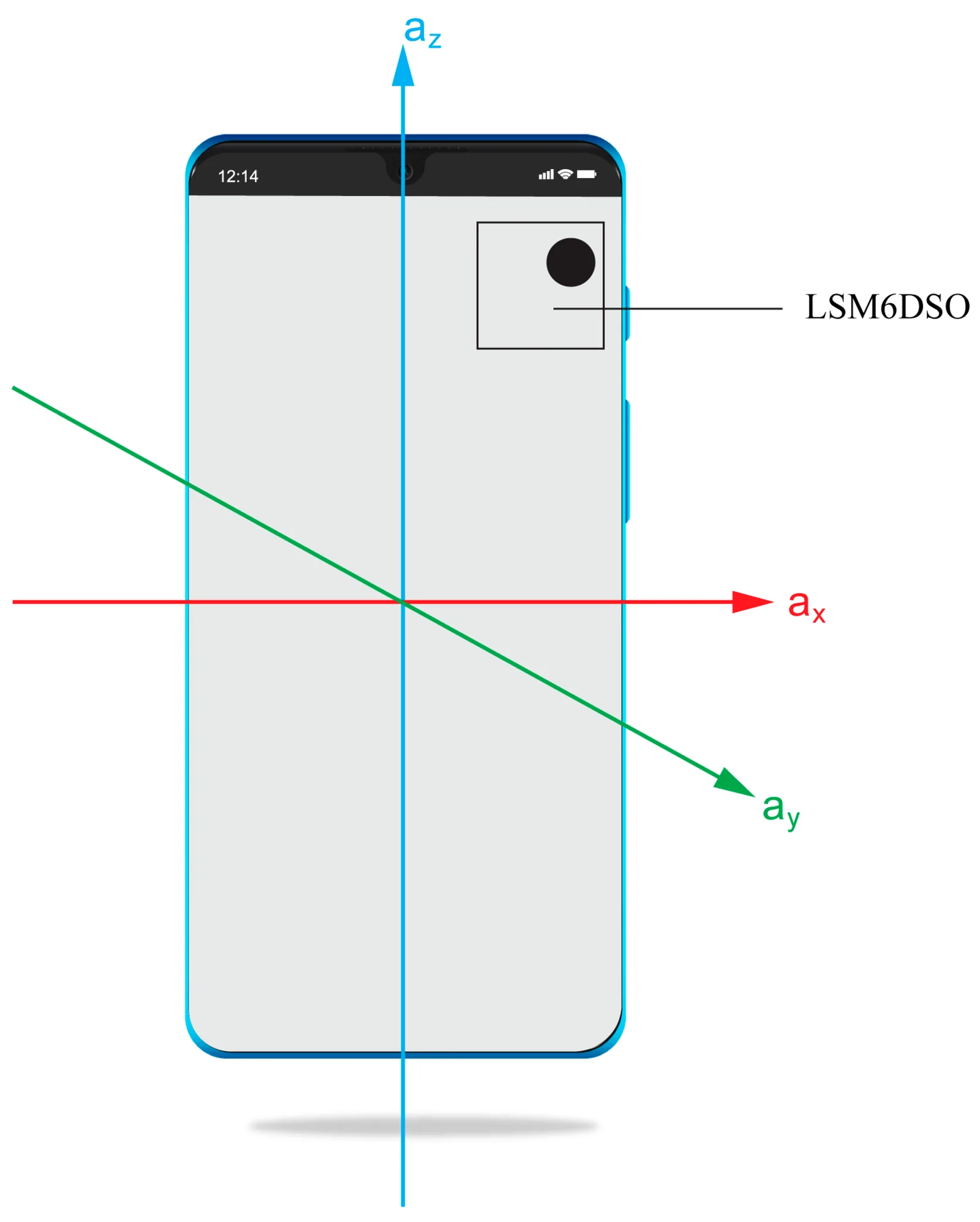
LSM6DSO coordinate system in smartphone, reproduced from the author M.N.R.’s MSc thesis [27].

The sensor axis alignment followed the SAE J1490 standard recommendations [3]. The vibrations in x, y, and z are denoted as accelerations (ax, ay, az). The coordinate system of LSM6DSO was later adjusted according to SAE J1490, as shown in Figure 6.

The MEMS output data rate was set to 512 Hz. Raw tri-axial acceleration was recorded at $f_{s,in}$ = 512 Hz to oversample the 0.5–80 Hz band. In line with ISO 15037-3 data acquisition, anti-alias attenuation at the Nyquist frequency was enforced so that the magnitude of all frequency components is reduced below the digital resolution while maintaining ≤ 0.05% attenuation over the band of interest. Accordingly, an IMU internal low-pass was set near 100–120 Hz, followed by a linear-phase FIR low-pass design; the passband is 0–80 Hz, ripple ≤ 0.1 dB, and stopband ≥ 100 Hz, with attenuation ≥ 60 dB. The filtered stream was then resampled to $f_{s,proc}$ = 256 Hz for spectral analysis.

### 2.5. Smartphone Mounting Position and Alignment Protocol

**Figure 7:**
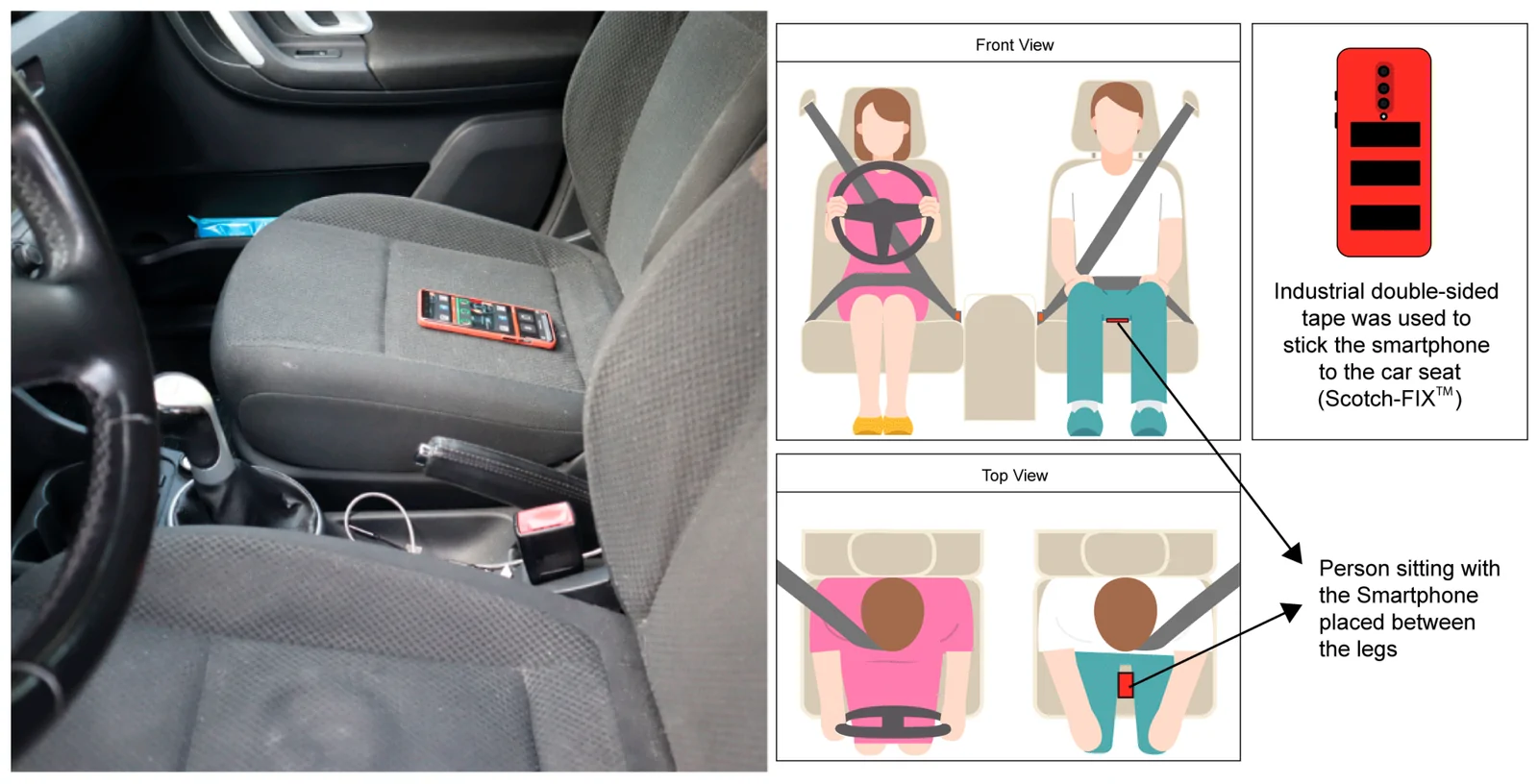
Mounting position of the smartphone in the car.

The smartphone was positioned on the supporting seat surface beneath the seated occupant, corresponding to the seat–occupant vibration measurement location specified in ISO 2631-1. For measurements on a resilient seat surface, ISO 2631-1 requires the transducer to be interposed between the person and the principal contact area of the seat. In the present study, the smartphone was secured using high-bond double-sided adhesive tape to prevent relative movement during testing, while a 72 kg occupant was seated in a normal posture, as shown in Figure 7. Although the measurement location followed the ISO 2631-1 seat–occupant interface principle, the smartphone and adhesive mounting arrangement differs from the standardized seat-pad accelerometer configuration used for formal whole-body vibration measurements. Accordingly, the present measurements are described as ISO 2631-1 aligned rather than formally ISO 2631-1 compliant.

The vehicle and road test conditions were selected with reference to the general passenger-car evaluation considerations described in ISO 15037-3. At the same time, vehicle loading was kept within the mass envelope defined by ISO 1176, specifically, M08, which limits the combined mass of the driver, occupant, and equipment to below 150 kg.

### 2.6. Ride Comfort Android Application

**Figure 8:**
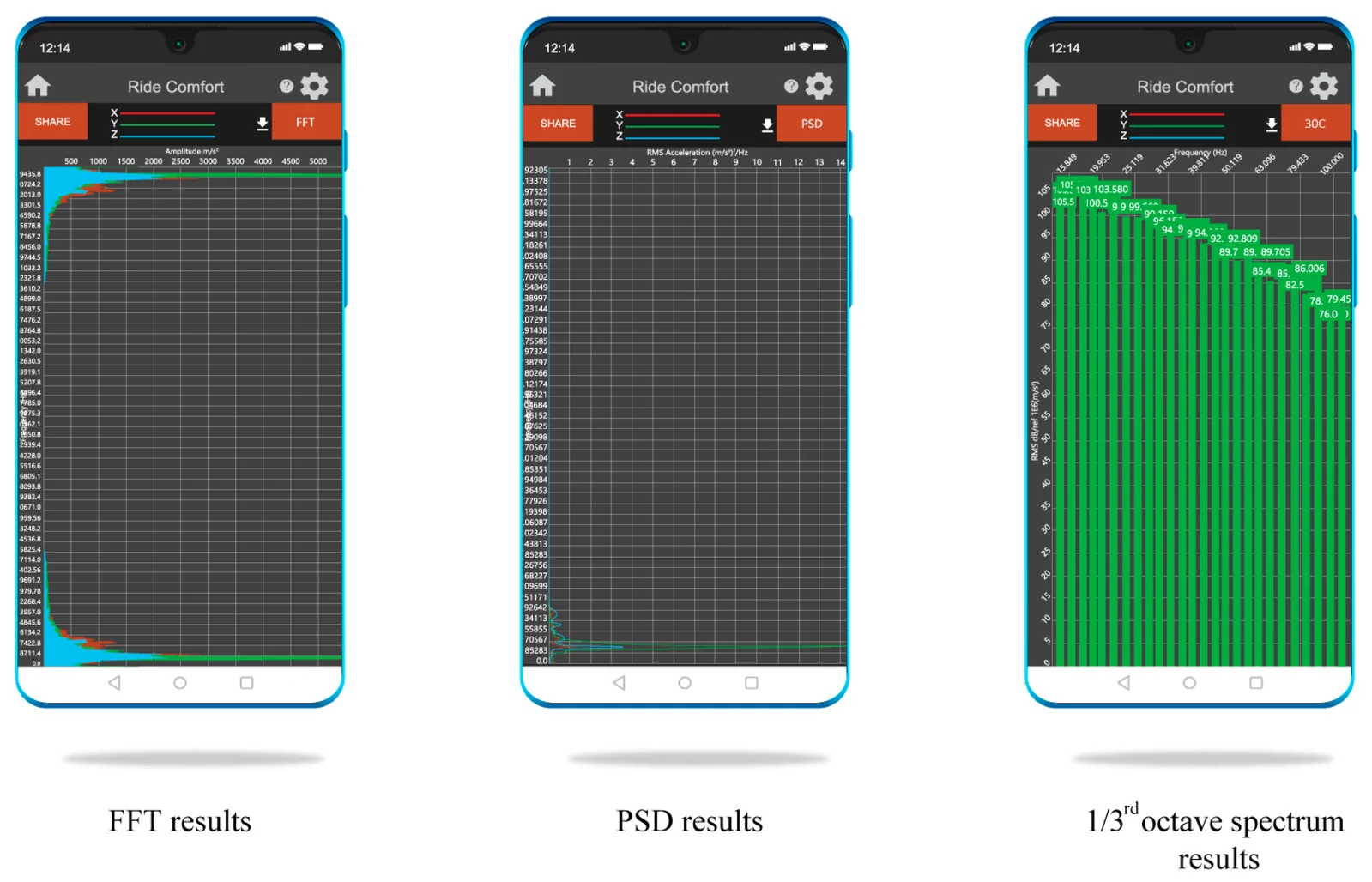
Illustrated Ride Comfort Android application with FFT, PSD, and 1/3rd-Octave spectrum.

A complete Android application was developed using Kotlin in Android Studio to access tri-axial raw vibration data from the LSM6DSO sensor and to perform post-computing for signal processing, including FFT, PSD, and the 1/3rd-Octave spectrum, as shown in Figure 8. Although the “Ride Comfort” application supports interactive visualization of graphs in real time, screenshots are not efficient for academic reporting. Consequently, the raw vibration data and computed data of FFT, PSD, and the 1/3rd-Octave spectrum are exported as a CSV file. All figures in this paper were then plotted again using Origin Lab Pro to enhance understanding and achieve publication quality.

The math functions are computed individually to avoid crashing the application. The basic requirement to install the application is that the operating system should be Android version 6 or higher.

## 3. Computation of Signal Processing Algorithms

**Figure 9:**
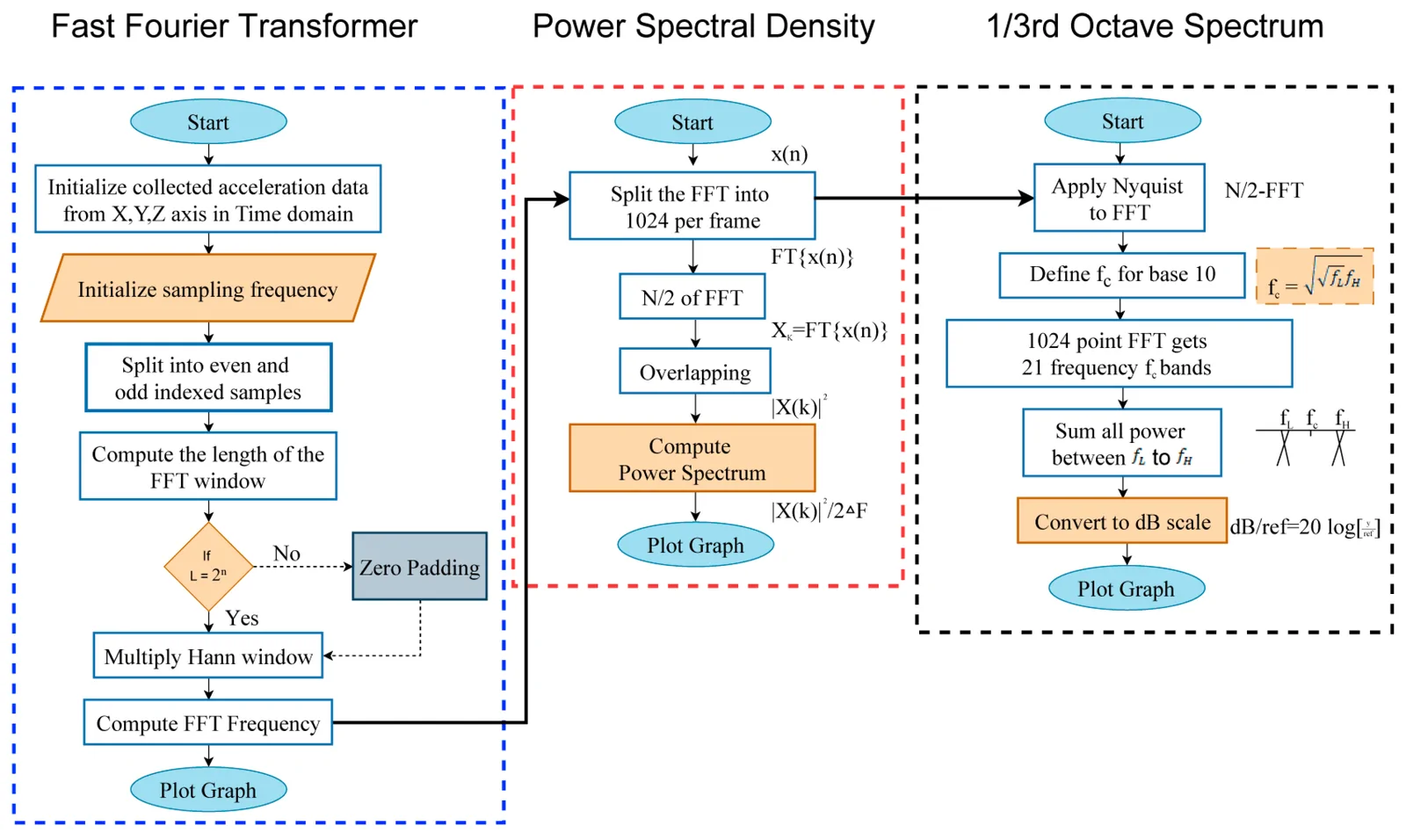
Flowchart representing FFT, PSD and 1/3rd-Octave spectrum algorithms.

This section explains the mathematical and computational algorithms behind the ride comfort application for FFT, PSD, and the 1/3rd-Octave spectrum. The flowchart shown in Figure 9 describes the computation of PSD, FFT, and the 1/3rd-Octave spectrum.

### 3.1. Fast Fourier Transform

There is a wide variety of FFT algorithms, with the Cooley–Tukey Fast Fourier Transform [28] being the most well-known. This specific FFT employs a butterfly diagram approach to compute the value of N, which is defined as 2^m^. Each operation involves both complex multiplication and complex addition [29].

Generally, the raw vibration data will be collected in the time domain as acceleration m/s^2^, and later, the sampling frequency will be computed using the raw vibration data [26,27].

(3) XK=∑n−0N−1xn exp(−j2πknN)

where $X_{K}$ is frequency domain samples, and $x_{n}$ is time domain samples.

(4) fs=Number of data or recordsSecond

FFT reduces N log2 N, instead of N2 operations, and later the sequence is split into two halves based on even (Gv) and odd (Hv) indexed samples to compute quicker [28].

(5) XK=∑v=0N2−1x2vexp−j2πkvN2+exp−j2πkN x2vexp−j2πknN2

Even indexed samples are:

(6) Gv=∑v = 0N2−1x2v+1exp− j2πkvN2

Odd indexed samples are:

(7) Hv=∑v = 0N2−1x2vexp− j2πkvN2

### 3.2. Computing the Length of the Window

**Figure 10:**
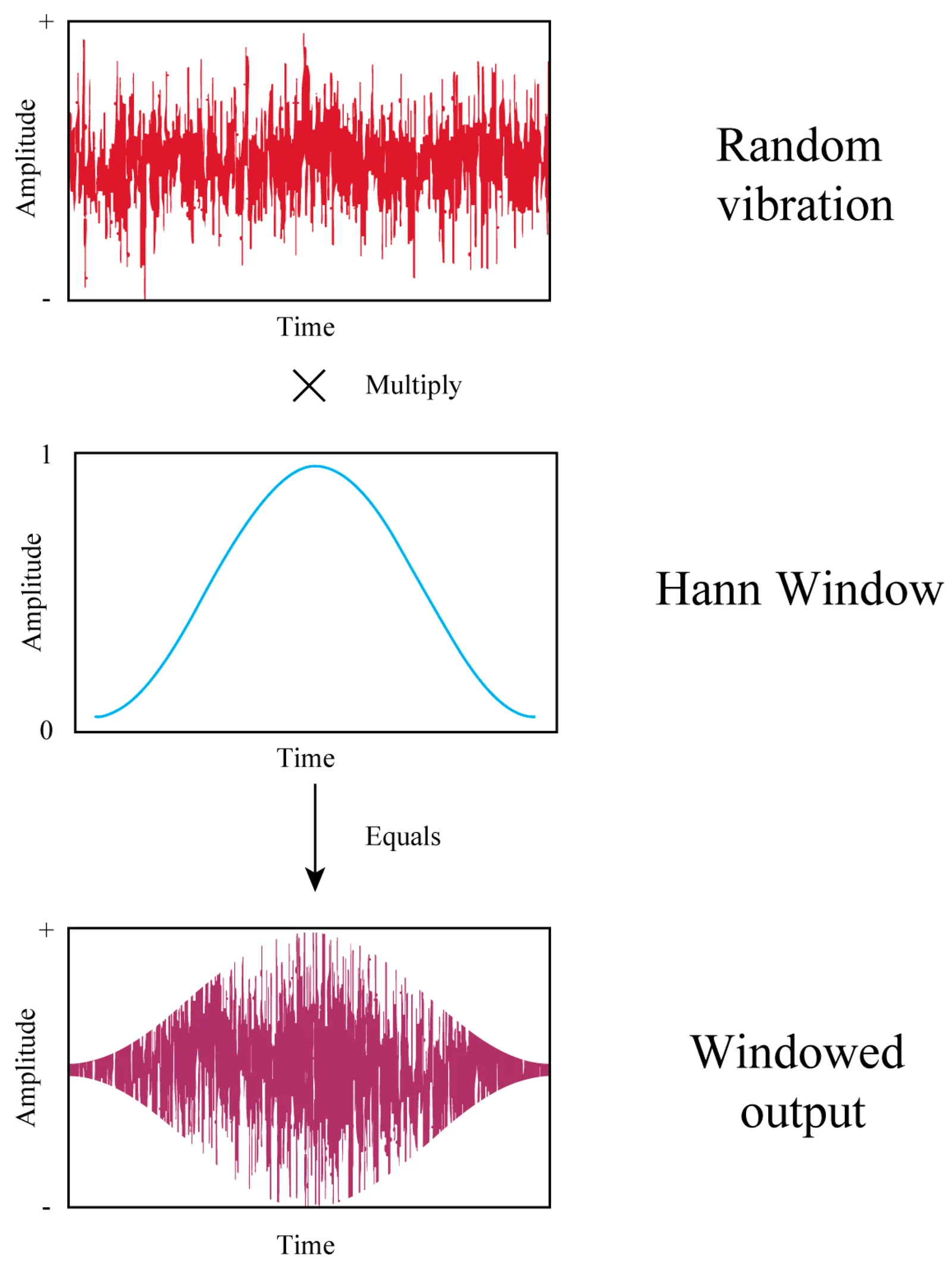
Use of Hann window in computation.

The length of the window is kept as a power of 2; when the length of the window is not a power of 2, zero padding will be used until the window reaches a power of 2 [29]. Zero padding is a method of adding empty or zero-valued samples to the end of the records until they reach a power of 2. For instance, when the recorded value or total samples is 900, the next closest value to the power of 2 is 1024. So, the zero-valued samples are added to truncate the closed value of 2^n^ [26,27]. The Hann window function is used for computing, as shown in Figure 10.

(8) wi=12−cos 2πin 

A weighting window function is applied over a segment, with its final sample denoted as n. This method helps with minimizing spectral leakage and significantly improves the overall frequency resolution. It is commonly employed in analyzing vibrations induced by road pavements [27,30,31].

### 3.3. Computing Power Spectral Density

Welch PSD is a widely used basic concept in signal analysis, which describes how signal power or a time series is distributed in different frequency components. PSD emphasizes frequency content instead of focusing on the amplitude of the signal over time and provides a clearer understanding of the main frequency components and energy distributions. It is particularly useful for analyzing random or complex signals such as vibration or noise data. PSD is usually calculated using a method such as an FFT or periodogram and plays a vital role in applications such as vibration detection [31,32,33].

The FFT is split into 1024 data per frame to compute the PSD from the FFT [27].

(9) $$X_{K}=FT\left\{X(n)\right\}$$

where K is 0, 1, 2, 3,…, (N/2)−1 [34,35].

(10) Power K=X(k)N2, k=0

And hence power (k) can be written as:

(11) Powerk=Xk2N22, k=1,2,3…., N2−

Power Spectral Density [34]

(12) PSD (K)=Power (k)∆f

where ∆f is the frequency difference [34,36]

(13) ∆f=1T

And the sampling frequency $f_{S}$ [34,36]

(14) fS=1Ts

**Figure 11:**
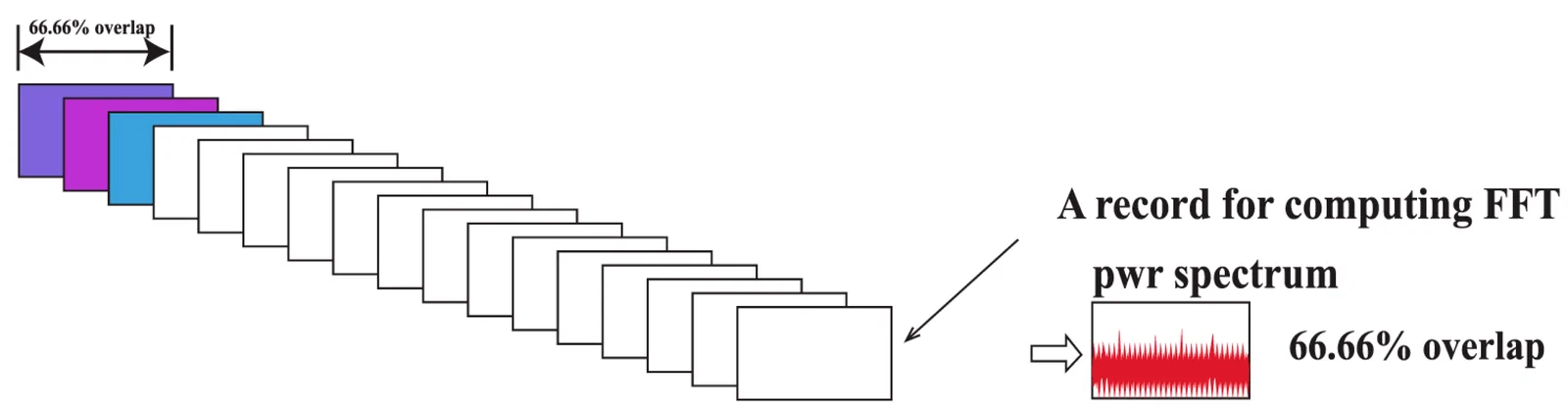
Illustration of 2/3rd, or 66.66%, overlapping used to compute PSD from FFT.

Accordingly, 66.66% or 2/3rd overlapping has been used in computing the power spectral density. Here, each frame overlapped with the previous frame to reduce jitter in time and to produce smoother time evolution with smaller hops, as shown in Figure 11. This provides better accuracy in results compared to 0% or 50% without moving to the 75% overlapping method. The only trade-off is that with higher correlation between segments, variance reduction is not linear with the number of segments [37,38,39].

### 3.4. Computing 1/3rd-Octave Spectrum

Octave spectrum filters are typically used to show the processed vibration data in decibel scale, which makes it easier to compare the obtained results with the standards. According to ANSI/ASA S1.6-2016, 1/3rd-octave bands are made as standard to measure noise with respect to SPL (Sound Pressure Level) and random vibration with respect to RMS in dB scale [40]. The reference values are given according to the SI units used to measure the data, as shown in Table 6.

In vibration analysis for the assessment of ride comfort, the number of available one-third-octave bands directly affects how accurately frequency content can be evaluated against human perception criteria. For ride comfort, standards such as ISO 2631 emphasize 1/3rd-octave band analysis because these bands align well with the human body’s frequency-dependent sensitivity to vibration. For example, with an *N* = 1024 FFT, the spectrum can be divided into 21 1/3rd-octave bands, providing sufficient resolution to capture key comfort-related frequencies. Table 7 links the FFT size to the count of standardized bands that can be resolved within the analysis range. This ensures that measured RMS vibration levels can be accurately assigned to the correct bands for comparison with ride comfort limits.

(15) dB Scale=dBref=20log10yref

For calculating the central frequency ($f_{c})$

(16) $$f_{c}=\sqrt{f_{H}f_{L}}$$

frequencies and lower frequencies.

(17) $$∆f=f_{H}-f_{L}$$

The 21 central frequencies of the 1/3rd- octave spectrum for 1024-point FFT from 0.8 Hz to 80 Hz.

### 3.5. Baseline Method for Comparative Evaluation

To assess the proposed Ride Comfort application against existing approaches, two published vibration–comfort evaluation methods were selected as computational baselines: the frequency-domain-weighted RMS approach of Zhao et al. [20] and the one-third-octave band method of Wawryszczuk et al. [1]. Both approaches were independently applied to the six existing vehicle test datasets. The comparisons were restricted to vertical acceleration (Y-axis), corresponding to the measurement direction used for the comfort assessment in the present study.

#### 3.5.1. Frequency-Domain-Weighted RMS Method

Zhao et al. [40] proposed a smartphone-based passenger comfort assessment method that uses frequency-dependent acceleration weighting. Their vertical weighting function is based on an approximation specified in GB 4970-1996. Following their frequency domain formulation, the weighted RMS acceleration was calculated as

(18) $$a_{w,Z}=\sqrt{\int_{0.5}^{80}G_{y}\left(f\right)\;{\left[W_{Z}\left(f\right)\right]}^{2}\;df}$$

where $G_{y}(f)$ is the acceleration PSD, and $W_{z}(f)$ is the vertical frequency-weighting function defined by Zhao et al. The comparison reproduces the vertical-axis component of their method rather than the complete three-axis evaluation framework.

#### 3.5.2. One-Third-Octave-Weighted RMS Method

Wawryszczuk et al. [1] employed an ISO 2631-1-based method in which vibration acceleration is evaluated within one-third-octave frequency bands. For the vertical direction, the weighted RMS acceleration is obtained as

(19) $$a_{w,W}=\sqrt{\sum_{i=1}^{N}{\left[W_{k}\left(f_{i}\right)\;a_{y,i}\right]}^{2}}$$

where $a_{y,i}$ is the unweighted RMS acceleration in the i-th one-third-octave band, $W_{k}\left(f_{i}\right)$ is the ISO 2631-1 vertical frequency-weighting factor at the band center frequency $f_{i}$, and N is the number of bands covering 0.8–80 Hz.

For the present implementation, the RMS acceleration in each band was obtained from the integrated spectral power using Equation (20)

(20) $$a_{y,i}=\sqrt{\int_{f_{i,L}}^{f_{i,U}}G_{y}\left(f\right)\;df}$$

where $f_{i,L}$ and $f_{i,U}$ denote the lower and upper frequency limits of the corresponding one-third-octave band.

#### 3.5.3. Comparative Assessment

Both baseline methods were applied to the same recorded acceleration signals used by the proposed Ride Comfort application. The raw acceleration records were mean-centered, and a one-sided PSD was estimated using a full-record periodogram. The weighted RMS results were subsequently compared using the percentage deviation using Equation (21)

(21) $$D_{m}=\frac{a_{w,app}-a_{w,m}}{a_{w,m}}\times 100\%$$

where $a_{w,app}$ represents the weighted RMS reported by the proposed application, and $a_{w,m}$ represents the corresponding baseline result from either method.

The comparison considers weighted RMS magnitudes, percentage deviations, and vehicle-comfort rankings within each road–speed condition. Only the vertical-axis computational components of the published methods were reproduced. Differences are therefore interpreted as differences in computational outcomes rather than evidence of superior measurement accuracy.

## 4. Results

The testing of the Ride Comfort application was carried out in the city of Prague, as mentioned in Section 2. Two tests were conducted using each car at speed, under the same road conditions. The supplementary dataset, including raw acceleration records, PSD, RMS, and octave band spectra from all test drives, is openly available on Zenodo [41].

### 4.1. Repeatability Test Using Skoda Fabia at an Average Speed of 90 km/h

Five tests were conducted consecutively on the same day to validate the “Ride Comfort” application’s reliability and repeatability on the E442 highway using a Skoda Fabia at a speed of 90 km/h.

#### 4.1.1. PSD Results at 90 km/h on Skoda Fabia

**Figure 12:**
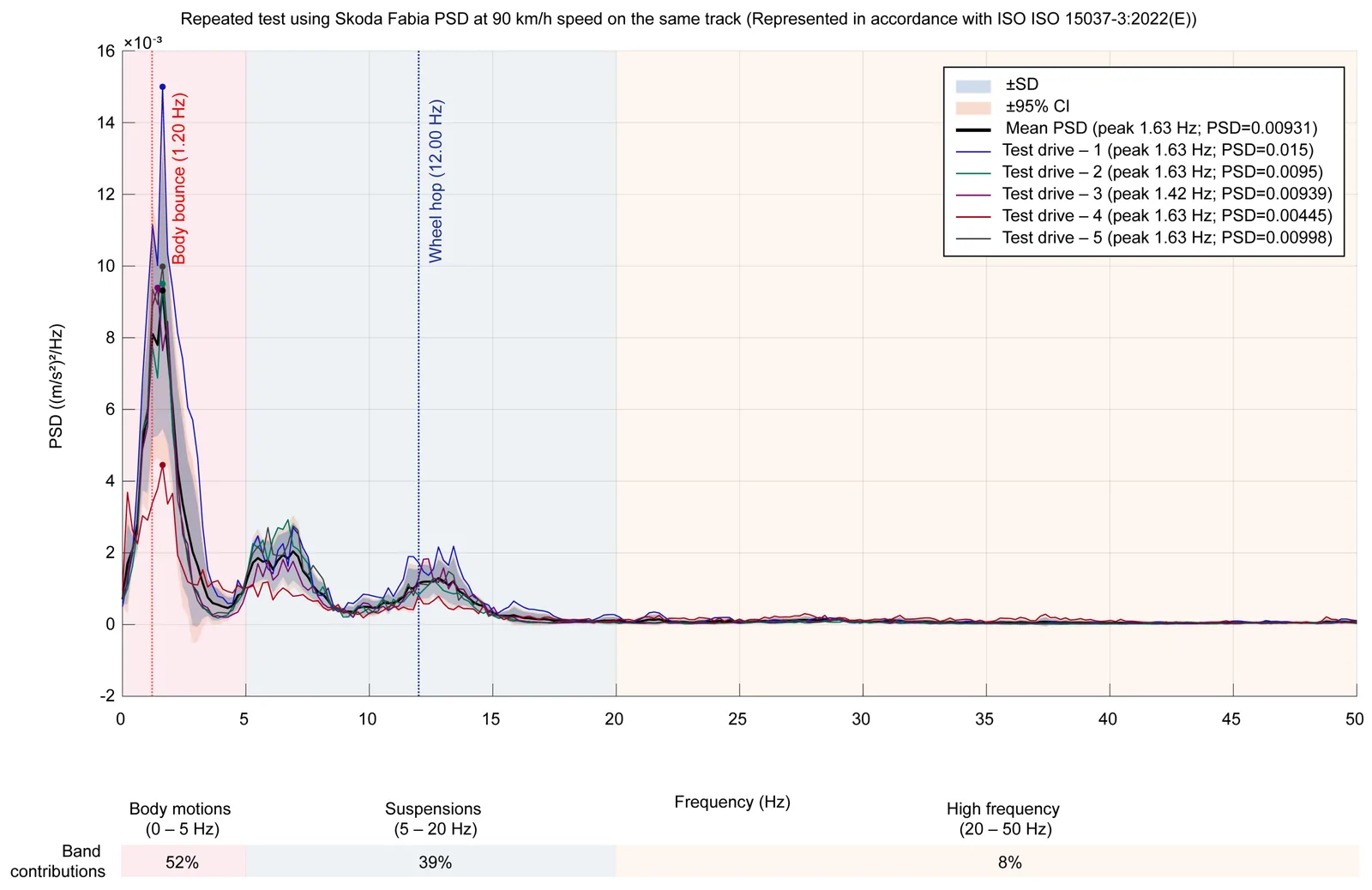
PSD results from all five tests at 90 km/h.

All runs exhibit a dominant low-frequency peak associated with body bounce near 1.6 Hz, which clearly shows four runs at 1.63 Hz and one run at 1.42 Hz, with similar secondary content between 5 and 20 Hz, commonly attributed to suspension dynamics, as shown in Figure 12. The mean PSD curve lies within a narrow standard-deviation band across 0.8–20 Hz, and the 95% confidence interval remains significantly close on each test, except at the very lowest frequencies, where road-influenced transients and speed micro-variations are most influential. The mean PSD remains inside a narrow ±SD range over 0.8–20 Hz, and the 95% confidence band is tight except below ~1 Hz, indicating low dispersion across runs and a precise estimate within the ISO 15037-3:2022 body motions (0–5 Hz) and suspension (5–20 Hz) bands. This stability is consistent with ISO 2631-1 practice, where steady-state ride vibrations of interest lie mainly below 20 Hz, and repeat measurements should report statistical descriptors of uncertainty. Overall, the statistics demonstrate ISO-aligned repeatability and operational reliability for the fixed road–vehicle–speed condition, with formal ISO compliance limited by the non-standard smartphone accelerometer rather than the analysis method.

#### 4.1.2. RMS Results at 90 km/h on Skoda Fabia

**Figure 13:**
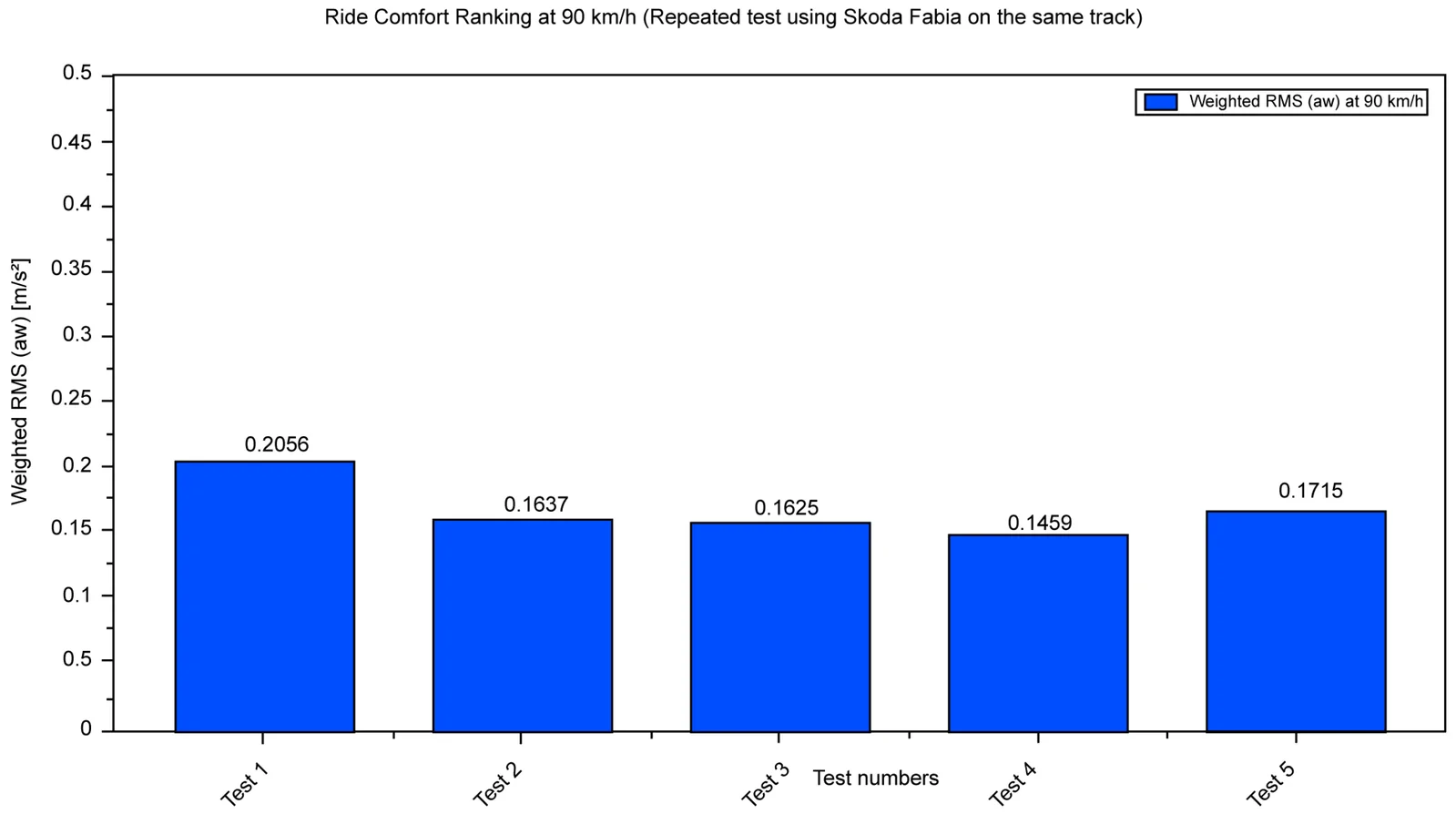
Frequency-weighted RMS acceleration (aw) for all five tests at 90 km/h using Skoda Fabia.

Five repeated runs on the same track (E442 highway) and speed yield ISO-weighted RMS (aw) values of 0.205 m·s^−2^, 0.163 m·s^−2^, 0.162 m·s^−2^, 0.145 m·s^−2^, and 0.171 m·s^−2^, as shown in Figure 13. All values fall well below the 0.315 m/s^2^ guidance level for “not uncomfortable” exposures in ISO 2631-1, indicating consistently low vibration severity for this fixed road–vehicle–speed condition.

### 4.2. Testing at an Average Speed of 30 km/h

Three tests were done to validate the “Ride Comfort” application in Prague at an average speed of 30 km/h using three different cars. The original screenshots of graphs from the Android application are provided in Appendix A.

#### 4.2.1. PSD Results at 30 km/h

**Figure 14:**
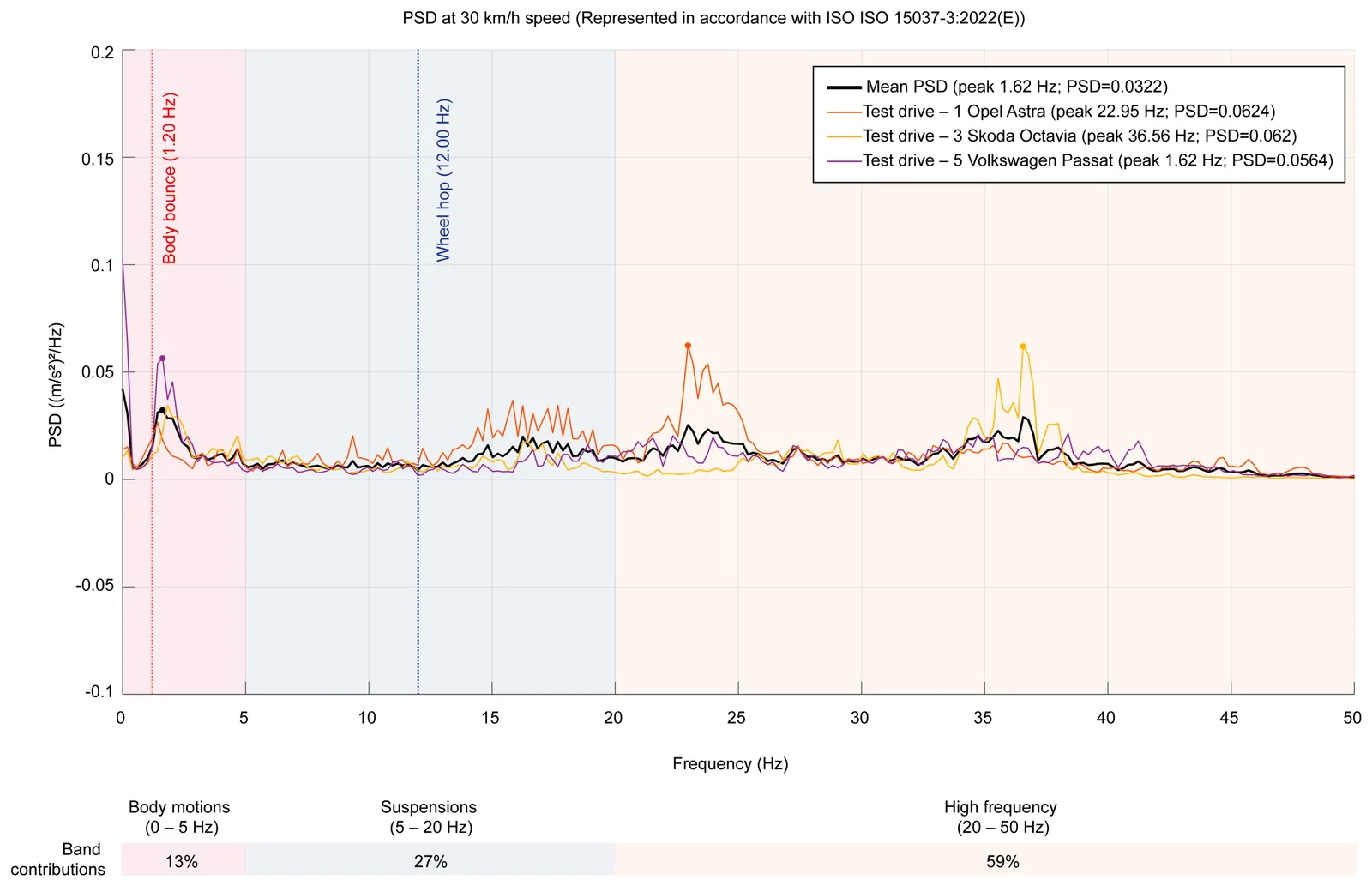
PSD results from all three tests at 30 km/h.

Figure 14 illustrates the results of the PSD magnitude in vertical acceleration (Y-axis) from all three tests carried out using three different vehicles at an average speed of 30 km/h. The Volkswagen Passat from test 1 has the peak PSD magnitude of 0.062 (m·s^−2^)^2^/Hz at the frequency of 22.95 Hz, while the Volkswagen Passat from test drive 5 has the lowest PSD magnitude of 0.056 (m·s^−2^)^2^/Hz at the frequency of 1.62 Hz. The Skoda Octavia from test drive 3 exhibits a powerful resonance at 36.5 Hz, with a peak value of 0.062 (m·s^−2^)^2^/Hz, indicating a higher sensitivity to low-frequency ride disturbances that significantly affect comfort perception. Energy partitioning reflects this behavior, that is, approximately 13% of total spectral energy lies in 0–5 Hz, 27% in 5–20 Hz, and 59% in 20–50 Hz. Below 20 Hz, the envelopes narrow, and the spectral shapes across vehicles become more alike, with a stable body-motions peak near 1.6 Hz.

#### 4.2.2. The 1/3rd-Octave Spectrum Results at 30 km/h

**Figure 15:**
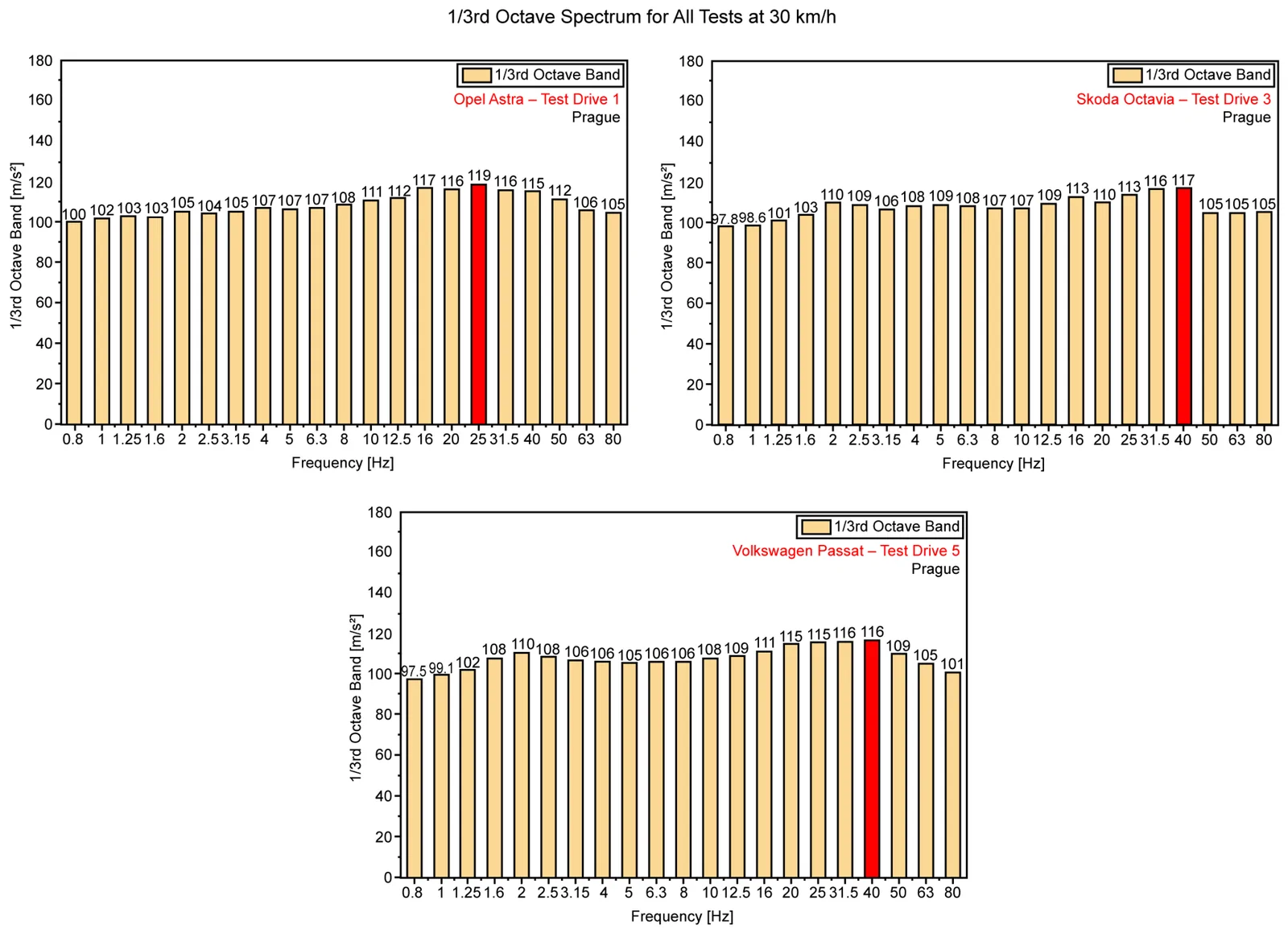
Illustration of 1/3rd-octave spectrum results from all three tests at 30 km/h.

Figure 15 presents the results of the 1/3rd-octave spectrum in vertical acceleration (Y-axis) from all three tests carried out using three different vehicles at an average speed of 30 km/h. The Volkswagen Passat from test drive 5 has the lowest band level among other cars, with a modest peak value of 116 dB re 1 × 10^−6^ m·s^−2^ at 40 Hz, with modest increases in the 20 to 25 Hz range, which indicates a mixture of mid- and high-frequency excitations. While the Opel Astra from test drive 1 conducted in Prague shows the maximum amplitude of 119 dB re 1 × 10^−6^ m·s^−2^ at 25 Hz, and also indicates pronounced resonance at the mid-frequency range. The Skoda Octavia from test drive 3 exhibits its dominant frequency at 40 Hz with the amplitude of 117 dB re 1 × 10^−6^ m·s^−2^, which clearly shows its vibration energy is mostly concentrated at higher frequencies compared to the Opel Astra.

#### 4.2.3. Ride Comfort Ranking with RMS (a_w_) for All Three Tests at 30 km/h

**Figure 16:**
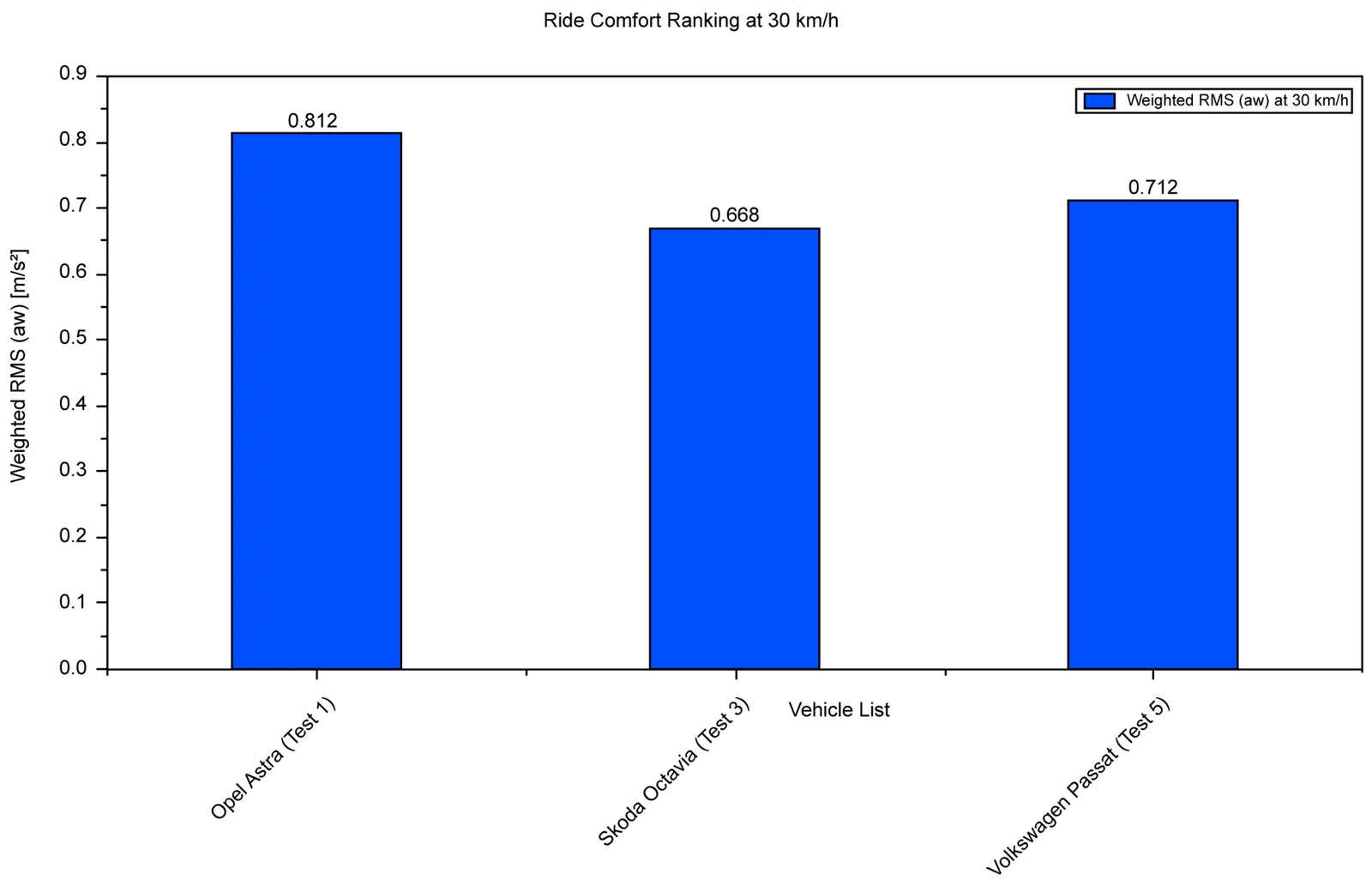
Frequency-weighted RMS acceleration (aw) for all three cars tested at 30 km/h.

Figure 16 presents the frequency-weighted RMS acceleration (aw) from all three cars tested at an average speed of 30 km/h and ranked according to the ISO 2631-1 ride comfort criteria. The Opel Astra and the Skoda Octavia recorded the highest and lowest values of 0.812 m·s^−2^ and 0.668 m·s^−2^, respectively, placing the Opel Astra from test drive 1 in the “uncomfortable” category. During the test drives 5, the Volkswagen Passat recorded an acceleration of 0.712 m·s^−2^, which places both the Skoda Octavia and Volkswagen Passat from test drive 3 and 5 in the “fairly uncomfortable” category.

### 4.3. Testing at an Average Speed of 50 km/h

Three tests were conducted to verify the “Ride Comfort” application in Prague while traveling at an average speed of 50 km/h in three distinct cars. The original screenshots of graphs from the Android application are shown in Appendix A.

#### 4.3.1. PSD Results at 50 km/h

**Figure 17:**
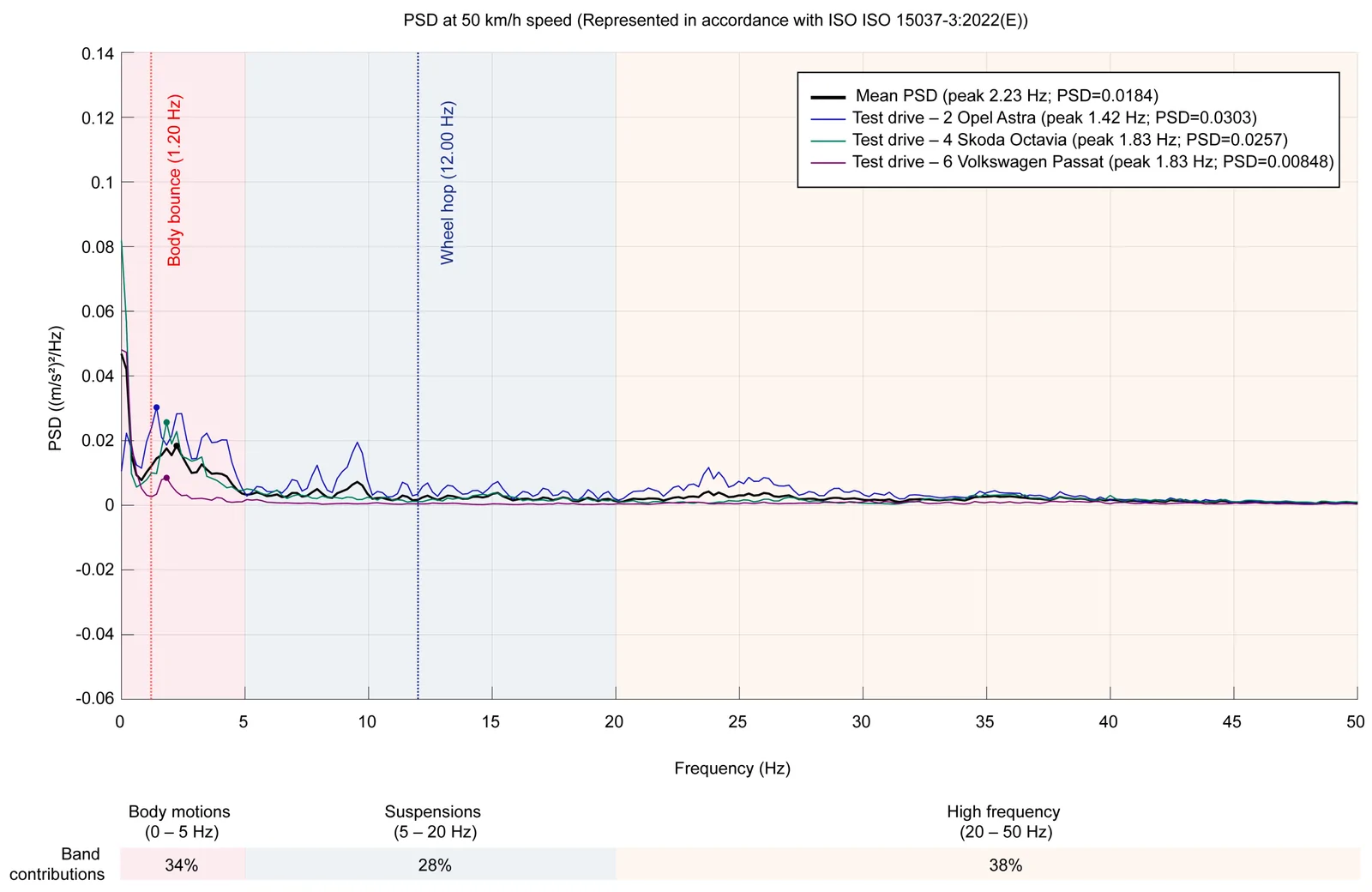
PSD results from all three tests at 50 km/h.

Figure 17 shows the PSD magnitude of vertical acceleration in the Y-axis for three tests conducted at an average speed of 50 km/h on different vehicles. The Opel Astra, from test drive 2, has a dominant peak of 1.42 Hz and a PSD magnitude of 0.030 (m·s^−2^)^2^/Hz. The Skoda Octavia in test drive 4 has a sharp resonance at 1.82 Hz and a maximum value of 0.026 (m·s^−2^)^2^/Hz. The Volkswagen Passat in Test Drive 6 exhibited the strongest low-frequency response at 0.92 Hz, with a PSD magnitude of 0.008 (m·s^−2^)^2^/Hz, and was sensitive to low-frequency driving inputs. The mean spectrum shifts slightly upward, with a primary peak near 2.23 Hz with PSD ≈ 0.0184, and the balance of energy becomes evener across bands, about 34% in 0–5 Hz, 28% in 5–20 Hz, and 38% in 20–50 Hz. All vehicles show pronounced body-motion content between 1.4 and 2.2 Hz, visible in the Opel Astra at 1.42 Hz, in the Skoda Octavia at 1.83 Hz, and in the Volkswagen Passat at 1.83 Hz. The dispersion bands are narrow from roughly 0.8 to 20 Hz, indicating closer agreement in the body motions and suspension ranges, with modest widening only in the upper band. This pattern is consistent with ISO 15037-3 band behavior; compared with the Track A condition at approximately 30 km/h, the Track B condition at approximately 50 km/h showed relatively stronger low-frequency content and reduced high-frequency dominance. Because the track and speed changed simultaneously, this difference cannot be attributed to speed alone.

#### 4.3.2. The 1/3rd-Octave Spectrum Results at 50 km/h

**Figure 18:**
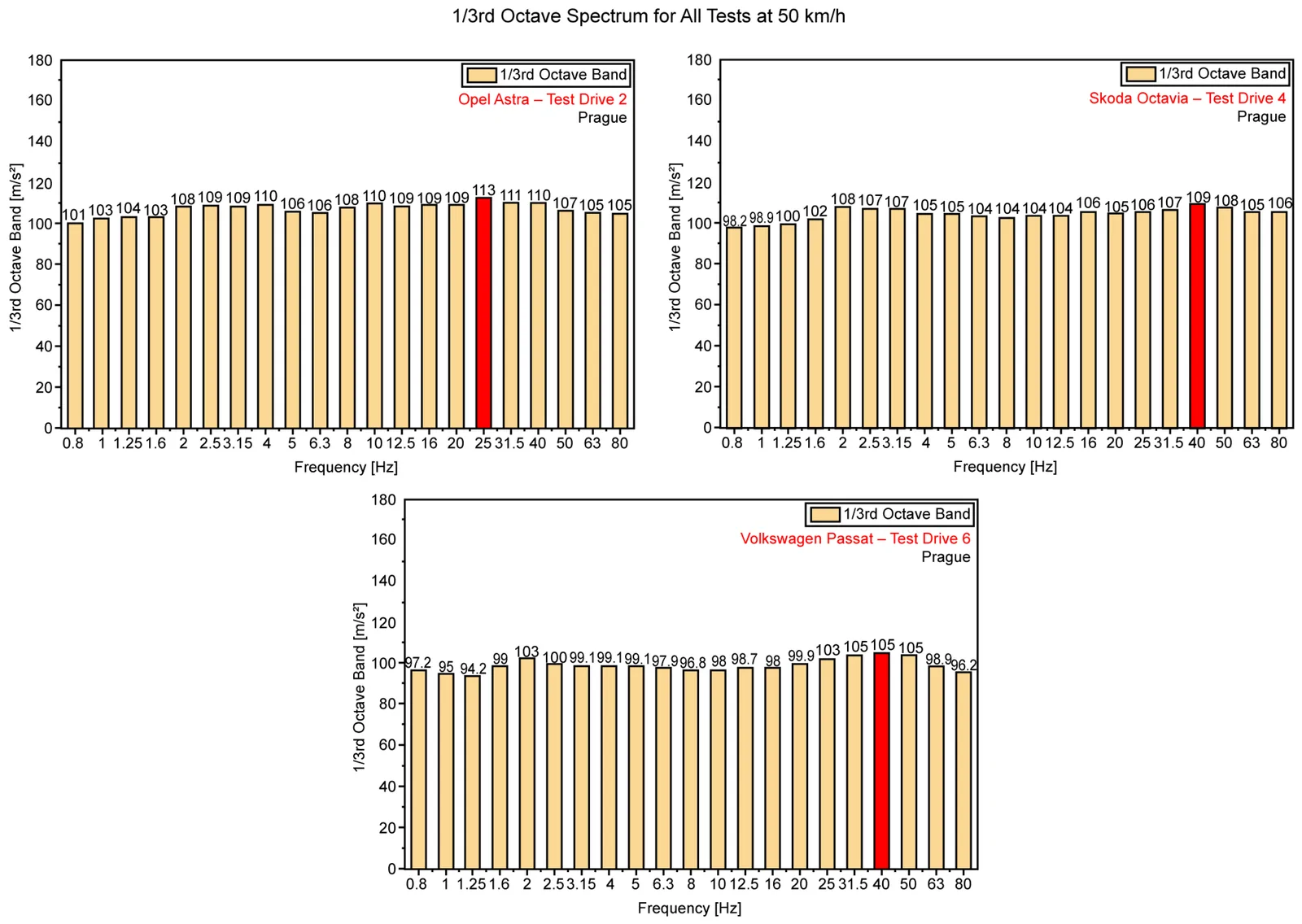
Illustration of 1/3rd-octave Spectrum results from all three tests at 50 km/h.

Figure 18 shows the 1/3rd-Octave spectrum of Y-axis vertical acceleration for the three tests conducted at an average speed of 50 km/h. The Volkswagen Passat from test drive 6 shows the lowest overall band level at 40 Hz, a peak of 105 dB re 1 × 10^−6^ m·s^−2^, generally a reduction in spectrum amplitudes, and smoother driving behavior at this speed. In contrast, the Opel Astra from test drive 2 demonstrated the highest peak at 113 dB re 1 × 10^−6^ m·s^−2^ at 25 Hz and demonstrated a strong mid-frequency resonance. The Skoda Octavia from test drive 4 shows a maximum amplitude of 109 dB re 1 × 10^−6^ m·s^−2^ at 40 Hz, and most vibration energy is concentrated in the higher frequency range. These results suggest that the Opel Astra tends to amplify at mid-frequency shocks, but the Skoda Octavia tends to concentrate on higher frequencies.

#### 4.3.3. Ride Comfort Ranking with RMS (aw) for All Three Tests at 50 km/h

**Figure 19:**
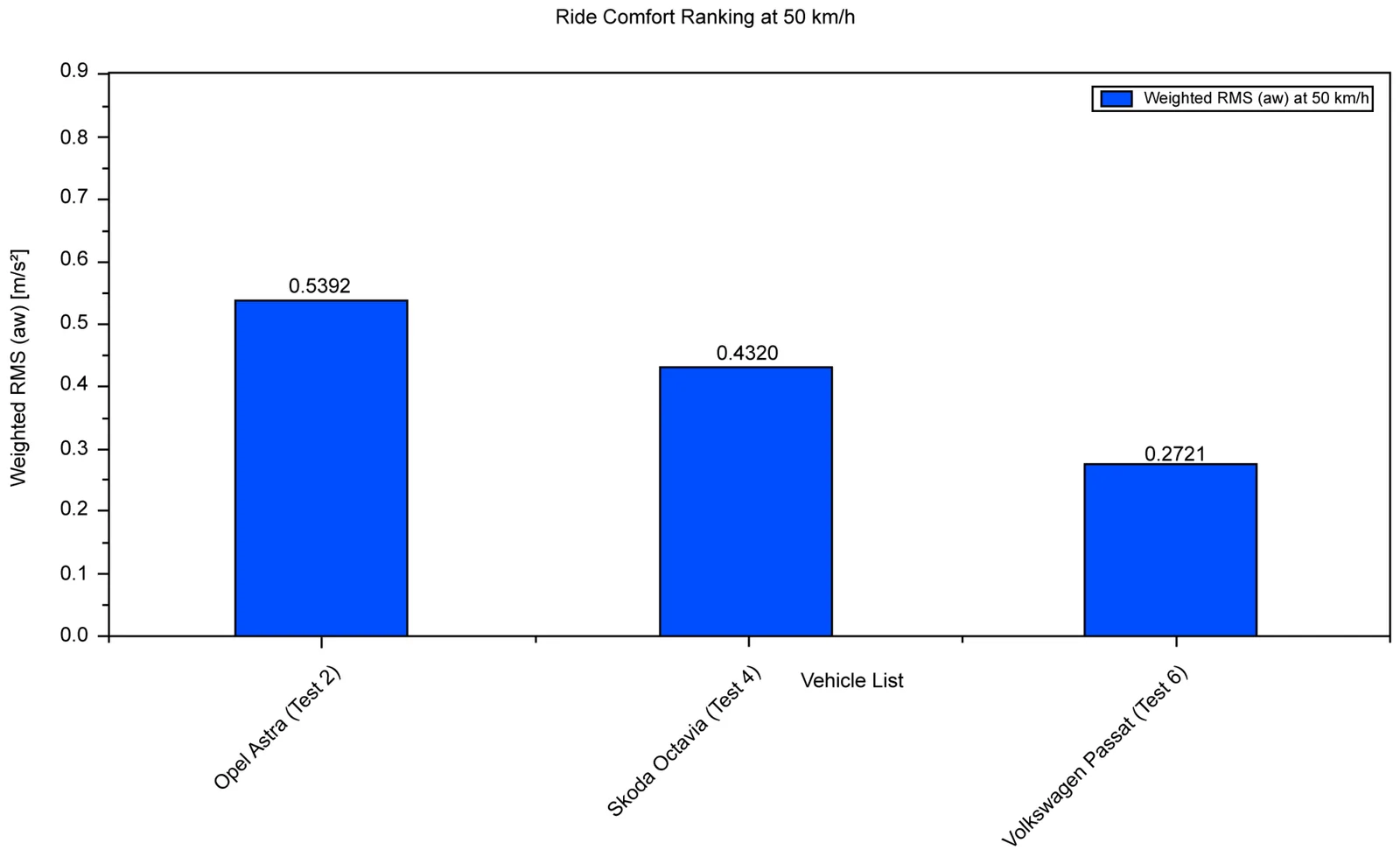
Frequency-weighted RMS acceleration (aw) for all three cars tested at 50 km/h.

Figure 19 shows the frequency-weighted RMS acceleration (aw) of three vehicles tested at an average speed of 50 km/h. The Opel Astra from test drive 2 conducted in Prague has a magnitude of 0.539 m·s^−2^ at the highest, placing it in the category of “fairly uncomfortable”. The Volkswagen Passat from test drive 6 achieved a lower value of 0.272 m·s^−2^, which falls under the “not uncomfortable” level. The Skoda Octavia from the 4th test drive achieved 0.432 m·s^−2^, which is classified as “a little uncomfortable”. The results indicate that the Volkswagen Passat at a driving speed of 50 km/h is the best, while the Astra is the lowest of the three vehicles. Table 8 shows the comparison of vehicle speed and obtained RMS results.

### 4.4. Comparison with Published Baseline Methods

The proposed Ride Comfort application was compared with the frequency domain method of Zhao et al. [20] and the one-third-octave method of Wawryszczuk et al. [1], using the same six acceleration datasets. Both methods were applied to the same six vibration datasets, following the computational procedures described in Section 3.5. Table 9 presents the frequency-weighted RMS acceleration values and percentage deviations obtained from the three approaches.

In addition to the comparison of frequency-weighted RMS acceleration magnitudes, the agreement in vehicle-comfort rankings among the three computational methods was evaluated. For each track, three pairwise vehicle comparisons were considered based on the weighted RMS acceleration values, with lower acceleration indicating better ride comfort. Under Track A conditions, the proposed Ride Comfort application achieved 66.7% pairwise ranking agreement with both Zhao et al. [20] and Wawryszczuk et al. [1], whereas complete agreement (100%) was observed under Track B conditions. Across both experimental conditions, the overall pairwise ranking agreement was 83.3% for each baseline method. In contrast, the two published baseline approaches exhibited 100% pairwise ranking agreement across both tracks. These findings demonstrate that although the proposed application generally reproduces the relative vehicle-comfort rankings obtained using existing computational approaches, differences in signal processing procedures may influence the ranking of vehicles under certain experimental conditions.

The proposed application exhibited mean absolute percentage deviations of approximately 10.6% relative to both methods. Individual deviations ranged from 1.6% to 19.9% for Zhao et al. and from 1.8% to 19.5% for Wawryszczuk et al. [1]. Under Track B conditions, all three methods produced identical vehicle-comfort rankings, with the Volkswagen Passat exhibiting the lowest weighted RMS acceleration. Under Track A conditions, the proposed application ranked the Skoda Octavia ahead of the Passat, whereas both baseline methods produced the reverse ranking.

To further evaluate the consistency between the published computational approaches, the absolute differences in frequency-weighted RMS acceleration between Zhao et al. [20] and Wawryszczuk et al. [1] were calculated for all six experimental datasets. The mean absolute difference between these two baseline methods was approximately 0.0033 m/s^2^, with individual differences ranging from 0.000 to 0.011 m/s^2^. This close numerical agreement indicates that the two baseline implementations produced similar weighted RMS acceleration estimates under the investigated conditions. In comparison, the proposed Ride Comfort application exhibited larger deviations from both baselines, particularly for the Opel Astra measurements. These differences highlight the sensitivity of weighted RMS acceleration estimates to the adopted computational procedures. However, because the effects of frequency weighting, spectral estimation, and band integration were not independently isolated, the observed deviations cannot be attributed to any single processing factor. Furthermore, agreement between computational methods does not establish absolute measurement accuracy without validation against calibrated reference instrumentation.

**Figure 20:**
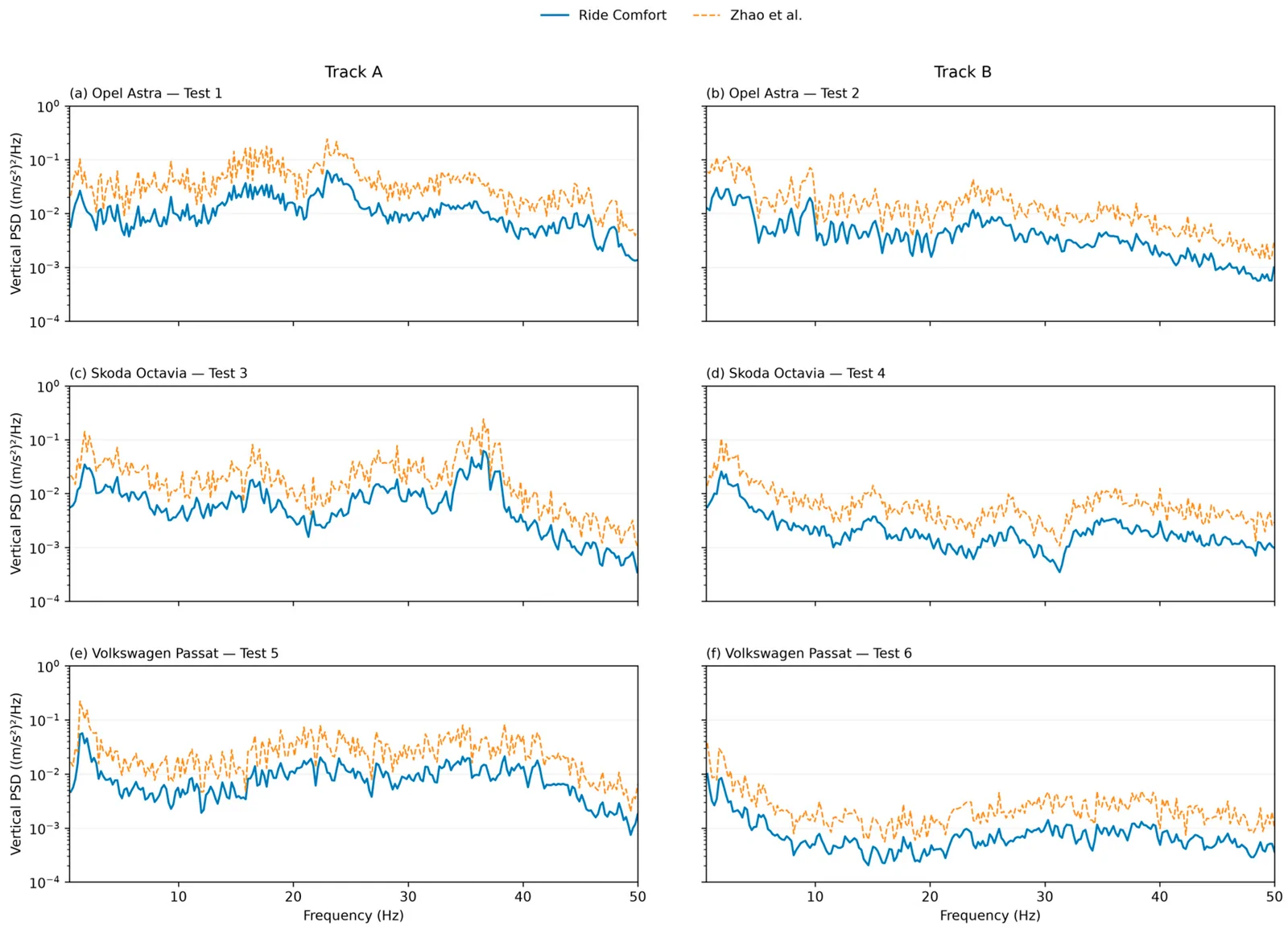
Comparison of vertical acceleration power spectral density obtained using Ride Comfort and the frequency domain approach of Zhao et al. [40]: (**a**,**b**) Opel Astra, (**c**,**d**) Skoda Octavia, and (**e**,**f**) Volkswagen Passat under Track A and Track B conditions, respectively.

Figure 20 compares the power spectral density obtained using the Ride Comfort application and the frequency domain approach based on Zhao et al. [20] for all six test drives. The proposed application employs Welch PSD estimation with 66.66% overlap, whereas the comparative implementation uses a full-record periodogram. Differences in spectral amplitudes and peak characteristics may therefore reflect the influence of spectral estimation and normalization procedures.

**Figure 21:**
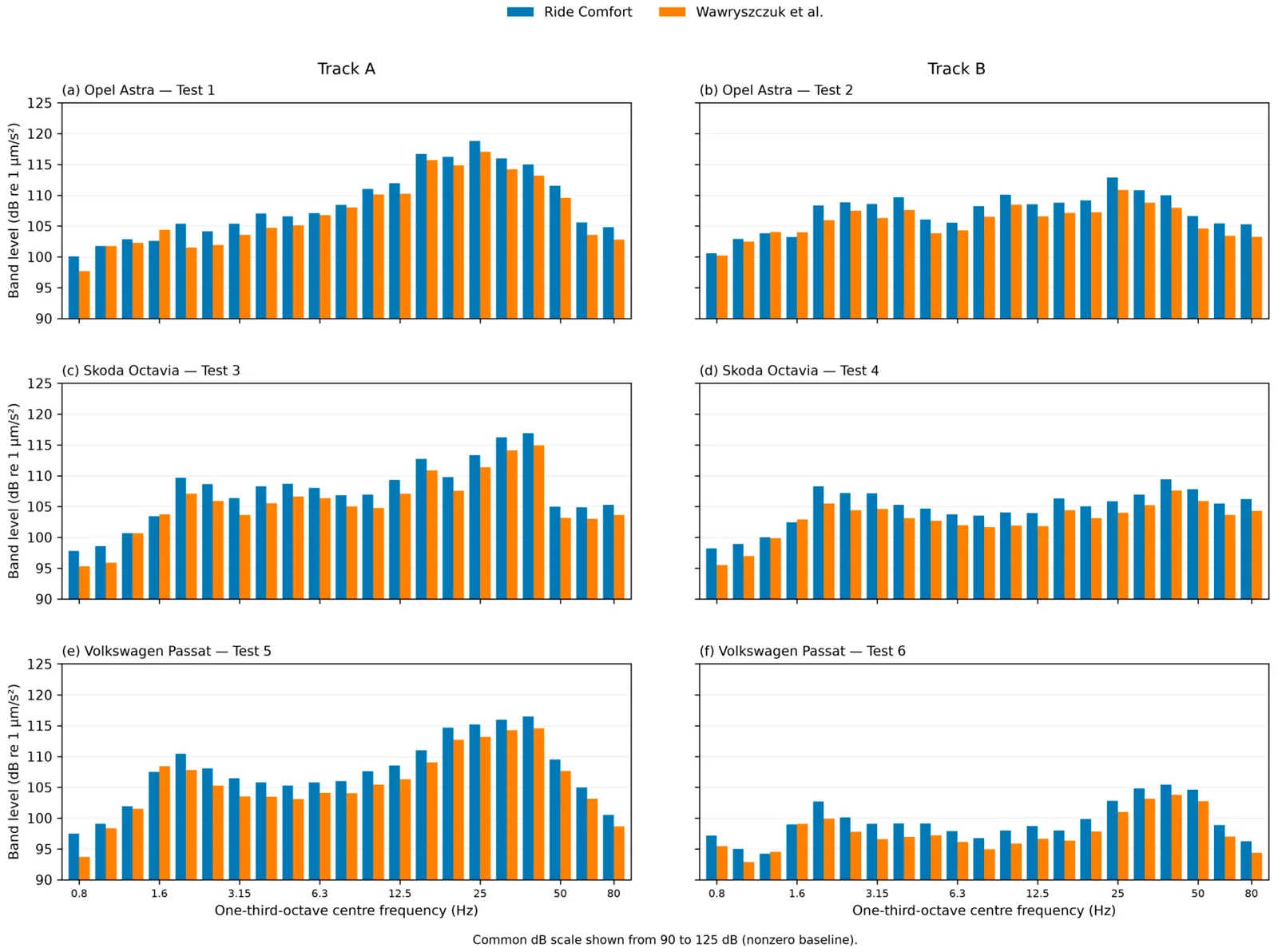
Comparison of one-third-octave acceleration spectra obtained using Ride Comfort and the band-based approach of Wawryszczuk et al. [1]: (**a**,**b**) Opel Astra, (**c**,**d**) Skoda Octavia, and (**e**,**f**) Volkswagen Passat under Track A and Track B conditions, respectively.

Figure 21 presents the corresponding one-third-octave spectral comparison between Ride Comfort and the band-based processing approach of Wawryszczuk et al. [1]. The results illustrate differences in vibration levels across the frequency bands, which may arise from variations in spectral estimation, band integration, and normalization. These frequency band characteristics provide additional context for interpreting the weighted RMS differences reported in Table 9.

Overall, the comparisons demonstrate that the proposed application provides vehicle-comfort assessments broadly consistent in ranking with the published computational approaches, despite differences in calculated vibration magnitudes. The observed deviations cannot be attributed to a single processing parameter and do not establish differences in measurement accuracy. The primary contribution of Ride Comfort remains the integration of spectral analysis, frequency-weighted RMS evaluation and comfort visualization within a standalone smartphone application.

## 5. Discussion

### 5.1. Accuracy of the Smartphone IMU Relative to a Standard Accelerometer

Translating laboratory whole-body vibration methods to daily-use smartphones requires establishing whether a smartphone-grade MEMS accelerometer can reproduce the output of a dedicated human vibration analyzer under on-road conditions. Agreement depends on three factors: mechanical coupling, calibration and scale, and preservation of signal quality within the analysis band. In this study, the device was rigidly coupled to the seat pan using a high-bond adhesive to eliminate interface slip and micro-motion, which would otherwise introduce spurious high-frequency content and bias the frequency-weighted RMS acceleration. Measurements were acquired with a seated occupant of mass 72 kg in a normal posture to replicate the loaded conditions assumed in ISO 2631-1. The smartphone’s factory calibration was used; gravity and static bias were removed in the software. Signal processing was designed to preserve low-frequency content and limit spectral leakage. On-device computation employed Welch power spectral density estimation with a Hann window and 66.6% overlap; the LSM6DSO anti-alias filter prevented spectral folding at the selected sampling rate; and orientation normalization aligned the device’s vertical axis with the seat. In the absence of a reference meter in this dataset, the reported aw values should be interpreted within an estimated ±5–10% uncertainty band based on prior studies [12,13,14]. Under rigid mounting, occupant loading, and disciplined band-limited processing, the smartphone reproduces the whole-body vibration indices that dominate ride comfort within this agreement.

### 5.2. Resonance Frequencies of Expected Vehicle Bands vs Obtained Spectra

Passenger-car vertical dynamics cluster into characteristic modal bands. The body bounce of the sprung mass is approximately 1.0 to 1.4 Hz for C and D segment cars. The wheel hop of the unsprung mass lies in the low teens, about 11 to 13 Hz. These bands are set by stiffness and mass and are not functions of speed; speed primarily scales excitation amplitude through the road wavelength content, as described by Gillespie [42].

Measured power spectral densities show a low-frequency peak near 1.0 to 1.3 Hz, corresponding to body bounce, and a second peak near 11 to 13 Hz, corresponding to wheel hop across the tested vehicles. The presence of a 72 kg occupant increases the effective sprung mass and shifts the bounce frequency slightly downward, consistent with the quarter car relation in which the natural frequency is proportional to the square root of the spring rate over mass, as detailed by Rajamani [43].

Additional speed invariant features appear between 25 and 50 Hz. The Opel Astra shows a pronounced feature near 25 Hz, whereas the Skoda Octavia and Volkswagen Passat are dominated near 40 Hz. Because these features do not translate with changes in speed, they are best interpreted as resonances of the seat and its mount, including the cushion, pan, sliders, rails, and coupling to the body in white, rather than responses to road wavelength. Controlled seat studies report resonance bands near 25–30 Hz and 45–50 Hz, with a lower band near 4–5 Hz, as reported by Qiu and Griffin [44]. Abdul Jalil and Griffin [45] further showed that seat–occupant vibration transmission is influenced by backrest inclination, seat pan inclination, and measurement location. Vibration characterizations also show that a nominal 26 Hz seat-only mode can shift toward 40 Hz when coupled to a nearby floor or body in white mode, consistent with the peaks near 40 Hz observed in heavier D segment vehicles.

Within ISO 2631-1, the W_k_ weighting emphasizes frequencies below approximately 8 Hz, so energy above 8 Hz contributes less to the frequency-weighted RMS aw than sub-8 Hz content. The higher bands between 25 and 50 Hz nevertheless shape subjective feel and can contribute to local discomfort during prolonged exposure, as discussed by Zuo and Nayfeh [46].

### 5.3. Interpretation of Results

The road surface and vehicle speed jointly determine the vibration excitation experienced by the vehicle and occupant. In the present experiments, however, these factors were not varied independently. Track A was tested at approximately 30 km/h, whereas Track B was tested at approximately 50 km/h. Therefore, the differences observed between these two conditions represent combined road–speed effects and cannot be attributed to vehicle speed alone.

Track A contained a larger proportion of cobblestone pavement, whereas Track B contained a larger proportion of asphalt. The stronger vibration content observed under the Track A condition is therefore consistent with the rougher surface characteristics of this route. In contrast, the Track B condition produced lower-weighted RMS acceleration for all three vehicles. Because the road surface and speed changed simultaneously, the present data cannot determine how much of this difference resulted from road roughness and how much resulted from vehicle speed.

The measured spectra nevertheless provide useful information about the vibration characteristics of the tested vehicles under each road–speed condition. Pronounced low-frequency components were observed in the body-motion range, together with vehicle-specific features between approximately 25 and 50 Hz. Differences in the suspension–tire–seat transmission path may contribute to these variations. The Astra showed a pronounced feature near 25 Hz, whereas the Octavia and Passat showed stronger features near approximately 40 Hz. These higher-frequency components were clearly visible in the spectral results, although the frequency-weighted RMS comfort metric is influenced more strongly by vibration in the lower-frequency range.

Consequently, the lower-weighted RMS values obtained on Track B at approximately 50 km/h should be interpreted as the response of the vehicles under that specific road–speed condition rather than as evidence that increasing speed improves ride comfort. A crossed experimental design in which both speeds are tested on both tracks would be required to separate the independent effects of road surface and vehicle speed.

Within-vehicle repeatability was evaluated on an identical 3 km E442 segment at a fixed speed of 90 km/h using the same Skoda Fabia for five runs. The ISO-weighted RMS aw values were 0.205, 0.163, 0.162, 0.145, and 0.171 m·s^−2^, giving a mean of 0.169 m·s^−2^, SD = 0.0221 m·s^−2^, COV = 13.0%, and a 95% CI of 0.142–0.197 m·s^−2^. The full protocol and test setup have been explained in Section 2 and Section 4 to support replication and reliability assessment.

### 5.4. Advantages of the Application

- The application executes the whole ISO 2631-1 chain on the smartphone, including computation of the RMS, Welch PSD with 66.66% overlap, and 1/3rd-octave spectrum, without the requirement of external post-processing.
- The application delivers real-time results with an application-specific 0–100 comfort index derived from ISO 2631-1 comfort-category boundaries for direct comparison across vehicles, routes, and speeds.
- The application is scalable and low cost for fleet screening, route benchmarking, and aggregated datasets with traceable raw and processed outputs as a CSV file.
- The application is portable and low cost since it requires only an Android smartphone, allowing rapid setup and broad deployment.
- The application demonstrated repeatability with summary statistics, means, standard deviations, and confidence intervals across repeated runs.

### 5.5. Comparative Discussion with Existing Ride Comfort Assessment Methods

The quantitative comparisons presented in Section 4.4 provide further insight into the computational consistency and methodological differences between the proposed Ride Comfort application and the published approaches of Zhao et al. [20] and Wawryszczuk et al. [1]. Although all three methods were evaluated using the same six experimental acceleration datasets, differences were observed in the resulting frequency-weighted RMS acceleration values. These findings indicate that the selection and implementation of vibration-processing procedures can influence the numerical assessment of passenger ride comfort, even when identical measurement data are used.

An important observation is the close agreement between the two published baseline implementations compared with their agreement with the proposed application. As shown in Table 9, the Zhao et al. [20] and Wawryszczuk et al. [1] approaches produced similar-weighted RMS acceleration estimates across the six experimental conditions, whereas the proposed application exhibited mean absolute percentage deviations of approximately 10.6% relative to each baseline. For example, the proposed application produced a weighted RMS acceleration of 0.812 m/s^2^ for the Opel Astra in Test 1, compared with 0.978 m/s^2^ obtained using both published baseline approaches. This difference corresponds to approximately 17.0% relative to the baseline values. The larger deviations observed for certain test conditions suggest that the computational consistency of the proposed application requires further investigation, particularly regarding the implementation of frequency weighting and spectral integration.

The methodological differences among the three approaches provide possible explanations for these variations. The proposed Ride Comfort application employs Welch power spectral density estimation using a Hann window with 66.66% overlap, followed by ISO 2631-1-aligned frequency weighting. In contrast, the comparative implementation based on Zhao et al. [40] adopts a frequency domain weighting formulation derived from GB 4970-1996, while the approach based on Wawryszczuk et al. [1] evaluates weighted vibration through one-third-octave frequency band integration. Furthermore, both baseline implementations in the present comparison use a full-record periodogram. Differences in weighting functions, spectral estimation, normalization, and frequency band integration may therefore contribute to the observed variations in weighted RMS acceleration. However, because these computational factors were not independently evaluated, their individual contributions cannot be conclusively established.

The comparison of vehicle-comfort rankings provides additional insight into the practical implications of these numerical differences. The proposed application achieved an overall pairwise ranking agreement of 83.3% with each published baseline method. Complete agreement was observed under Track B conditions, whereas the relative ordering of the Skoda Octavia and Volkswagen Passat differed under Track A conditions. This finding indicates that variations in computational processing may affect comparative ride comfort assessments, particularly when vehicles exhibit relatively similar vibration levels. Therefore, agreement in vehicle ranking should be considered alongside differences in absolute weighted RMS acceleration when interpreting the consistency of the methods.

From an implementation perspective, the main contribution of the proposed application differs from the primary objectives of the two published approaches. Zhao et al. [40] investigated smartphone-based passenger comfort assessment using frequency domain weighting, whereas Wawryszczuk et al. [1] employed conventional vibration measurement and one-third-octave analysis. The proposed Ride Comfort application integrates tri-axial acceleration acquisition, FFT, Welch PSD estimation, one-third-octave spectral analysis, frequency-weighted RMS computation, comfort visualization, and CSV data export within a standalone Android application. Consequently, its contribution lies primarily in providing an integrated on-device measurement and analysis workflow rather than introducing a fundamentally new vibration-weighting algorithm.

Nevertheless, the comparative results should not be interpreted as evidence of superior measurement accuracy or formal ISO 2631-1 compliance. Since all three computational approaches were evaluated using acceleration data acquired from the same smartphone, the comparisons assess differences in processing outcomes rather than independent sensor accuracy. Validation against a calibrated reference vibration analyzer, together with controlled investigations of the individual signal processing parameters, would be necessary to establish measurement agreement and identify the sources of computational differences. Overall, the comparative evaluation demonstrates the practical feasibility of the proposed integrated smartphone-based assessment approach while identifying important considerations for its further validation and methodological refinement.

### 5.6. Limitations and Future Work

- **Dataset breadth and repeatability:** The dataset spans a small number of vehicles and tracks at speeds of 30, 50, and 90 km/h, so broader validation across additional cars, speeds, and road surfaces remains a limitation for generalizability. A follow-up campaign is planned across additional vehicle segments and surfaces, extending the speed envelope and targeting ≥5 repeated runs per condition to quantify between-vehicle and between-surface variance.
- **Reference benchmarking:** The measurement was not performed for this dataset using an ISO-compliant human vibration meter. Prior studies report agreement within 5–10% during measurements [12,13,14]. A complete Bland Altman analysis, including bias and limits of agreement, and a coherence assessment of the measurement chain remain future work.
- **Mounting and contact quality:** The phone was rigidly attached to the seat, and measurements were obtained with a seated 72 kg occupant. No loss-of-contact events were detected. Variability in mounting parameters, including adhesive stiffness, pad thickness, and seat contour, can bias the high-frequency content. A comprehensive sensitivity study remains future work.
- **Hardware specificity:** Measurements were taken using a smartphone equipped with an LSM6DSO IMU at a fixed ODR and anti-alias filter setting. While the pipeline generalizes, sensor noise floors, scale factors, and anti-alias responses differ across devices; minimal per device calibration may be required.
- **Practical deployment and scalability:** Large-scale deployment is limited by user variability and device differences; the app does not record GPS data or driver details, and metadata is restricted to the car brand and model, vehicle registration number, and a manually entered country to infer left- or right-side driving, with no crowdsourcing pipeline. Future work will add a mounting and orientation checklist and automated pre-test quality control, extend metadata with privacy-preserving GPS segment identifiers and pseudonymous session IDs, and enable opt-in crowdsourcing with coarse spatial tiling, on-device checks, and server-side validation to support reproducible aggregation, cross-vehicle comparisons, and route-level comfort maps.
- **Reference benchmarking:** The experimental measurements were not validated simultaneously against a calibrated human vibration analyzer. Although the proposed application was compared with two published computational approaches, these comparisons assess processing outcomes rather than absolute sensor accuracy. Validation against a reference seat-pad accelerometer remains necessary to establish measurement agreement and formal ISO compliance.
- **Computational method comparison:** The proposed application showed mean absolute percentage deviations of approximately 10.6% relative to both published baselines. These differences result from frequency-weighting functions, PSD estimation, spectral normalization, and frequency band integration. Since these factors were not independently isolated, their individual contributions to the observed deviations remain undetermined. Future work will investigate these processing parameters and their influence on weighted RMS estimation.

## 6. Conclusions

This work developed and evaluated a smartphone ride comfort Android application named Ride Comfort. The app acquires three-axis acceleration from the on-board LSM6DSO sensor and completes the full pipeline on the device. Welch power spectra are computed with 66.66% overlap, ISO 2631-1 Wk weighting is applied over 0.5 to 80 Hz, 1/3rd-octave bands are formed, and the results are shown as weighted RMS aw and a clear comfort index from zero to one hundred percent. Data and results can be exported as CSV files for external analysis. The processing conventions follow ISO 2631-1, and the band interpretation follows ISO 15037-3. Instrumentation is smartphone-based; therefore, the study is ISO aligned rather than formally ISO compliant.

Field tests in Prague covered three vehicles under two road–speed conditions: Track A at approximately 30 km/h and Track B at approximately 50 km/h. The tool differentiated the ride comfort responses of the three vehicles under these test conditions. On Track A at approximately 30 km/h, the Opel Astra recorded a_w_ = 0.812 m·s^−2^ with an extended comfort rating of 67.67% and was classified as uncomfortable. The Skoda Octavia measuring a_w_ = 0.668 m·s^−2^ and the Volkswagen Passat measuring a_w_ = 0.712 m·s^−2^ were classified as “fairly uncomfortable”.

On Track B at approximately 50 km/h, the Opel Astra measured a_w_ = 0.539 m·s^−2^ and remained “fairly uncomfortable”. The Skoda Octavia measured a_w_ = 0.432 m·s^−2^ and was categorized as “little uncomfortable”. The Volkswagen Passat achieved aw = 0.272 m·s^−2^ and was “not uncomfortable” with an extended comfort rating of 97.15%. The lower-weighted RMS values observed under the Track B condition reflect the combined influence of road surface and vehicle speed. Because these factors were not varied independently, the present results should not be interpreted as evidence of an isolated speed effect.

These differences are also reflected in the power-spectral distributions across the ISO 15037-3 frequency bands; however, because the track and speed varied simultaneously, the observed spectral changes cannot be attributed to speed alone.

Within-vehicle repeatability was assessed on an identical 3000 m segment of the E442 highway at 90 km/h using the same Skoda Fabia for five runs. The weighted RMS values were 0.205, 0.163, 0.162, 0.145, and 0.171 m·s^−2^. The mean was 0.169 m·s^−2^, the standard deviation was 0.0221 m·s^−2^, and the coefficient of variation was 13%. The 95% CI for the mean ranged from 0.142 to 0.197 m·s^−2^. The mean spectrum stayed within a narrow ±1 SD envelope from 0.8 to 20 Hz, and the confidence band around the mean was tight except below about 1 Hz. The spectral shape was therefore stable across the body motions and suspension ranges defined by ISO 15037-3, which supports repeatability and operational reliability under fixed road, vehicle, and speed conditions.

To further evaluate the proposed method, the six experimental datasets were compared with the frequency domain approach of Zhao et al. [20] and the one-third-octave method of Wawryszczuk et al. [1]. The proposed application exhibited mean absolute percentage deviations of approximately 10.6% relative to both baseline methods (Table 9). All three methods produced identical vehicle-comfort rankings under Track B conditions, while differences in the Octavia and Passat rankings were observed under Track A conditions. The PSD and one-third-octave comparisons (Figure 20 and Figure 21) further illustrated variations associated with the adopted spectral-processing procedures. These deviations may arise from differences in frequency weighting, spectral estimation, and band integration, but do not establish differences in measurement accuracy. The comparative analysis supports the assessment of Ride Comfort against published computational approaches while highlighting its integrated on-device implementation.

In summary, this work shows that a smartphone can run the entire ISO-aligned measurement and analysis chain and report ride comfort metrics in real time, with no external post-processing. The novelty lies in a complete on-device implementation that records tri-axial acceleration, computes Welch spectra with 66.66% overlap, applies ISO 2631-1 Wk weighting and 1/3rd-octave bands, and outputs a deterministic comfort index that is directly comparable across runs and vehicles. A within-vehicle repeatability check at 90 km/h on a fixed route showed stable spectra and a modest spread in weighted RMS, supporting operational reliability under controlled conditions. Next steps are to enlarge the dataset across vehicles, speeds, surfaces, and loads; to benchmark against a calibrated seat pad accelerometer at the human–seat interface; and to study mounting sensitivity, occupant coupling, and links to reported comfort.

## Figures and Tables

**Table 1:** Methodological comparison of vibration-based ride comfort and road condition assessment approaches.

Distinction from Ride Comfort	Processing and Validation	Analysis/Metric	Primary Objective	Study
Emphasizes agreement with a reference instrument rather than an integrated spectral analysis workflow	Smartphone measurements compared with a dedicated human vibration analyzer	ISO 2631-1-based weighted RMS	Assess smartphone suitability for vehicle vibration measurement	Cargnin et al. [12]
Data-driven ride quality monitoring rather than a combined on-device FFT/PSD/octave display	Shaking table and on-road evaluation of a smartphone sensing framework	Learning-based ride quality assessment with ISO-based comfort comparison	Monitor urban road ride quality using smartphone sensing	Guo et al. [13]
Evaluates pavement condition, not ISO-weighted passenger comfort	Android smartphone; road condition detection evaluation	Accelerometer/gyroscope-based road condition classification	Identify road conditions from smartphone motion signals	Allouch et al. (RoadSense) [18]
The same integrated FFT, Welch PSD and one-third-octave workflow is not established	Smartphone-based passenger comfort assessment	Inertial sensor processing for passenger comfort evaluation	Evaluate passenger comfort using a smartphone application	Machaj et al. [19]
Targets pavement roughness rather than ISO-weighted passenger comfort	Vehicle-mounted smartphone with model-based roughness estimation	Vehicle-dynamics modeling and smartphone acceleration	Estimate road roughness using a low-cost smartphone	Rana and Asaduzzaman [15]
Combines smartphone sensing with supporting infrastructure rather than the same standalone spectral interface	Smartphone-based computation with an embedded server for display and alerts	Frequency domain weighting and weighted RMS	Assess bus passenger vibration comfort using crowdsourced data	Zhao et al. [20]
Conventional measurement/analysis configuration rather than smartphone-only implementation	Dedicated vibration instrumentation with external analysis	One-third-octave analysis and ISO-weighted vibration assessment	Assess passenger ride comfort in metro cars	Wawryszczuk et al. [1]
Acquisition, processing, comfort visualization and CSV export directly on the smartphone	Standalone Android application; six vehicle tests and five repeatability runs	FFT, Welch PSD (66.66% overlap), one-third-octave spectrum, ISO-aligned weighted RMS	Integrate passenger-car vibration analysis and comfort evaluation	**Proposed Ride Comfort**

**Table 2:** ISO 2631-1 standard guidelines for levels of comfort [22].

Level of Vibration Magnitude	Comfort Level
Less than 0.315 m·s^−2^	Not uncomfortable
0.315 to 0.63 m·s^−2^	A little uncomfortable
0.5 to 1 m·s^−2^	Fairly uncomfortable
0.8 to 1.6 m·s^−2^	Uncomfortable
1.25 to 2.5 m·s^−2^	Very uncomfortable
Greater than 2 m·s^−2^	Extremely uncomfortable
Level of vibration magnitude	Comfort level

**Table 3:** Details of test vehicles, test numbers, and test speeds.

Test Vehicle	Vehicle Segment According to the European Classification	Test Number	Approximate Speed	Test Duration	Track
Opel Astra	C—Segment	1	30 km/h	~61.8 s	A
		2	50 km/h	~61.8 s	B
Skoda Octavia	C—Segment	3	30 km/h	~60.96 s	A
		4	50 km/h	~61.2 s	B
Volkswagen Passat	D—Segment	5	30 km/h	~61.02 s	A
		6	50 km/h	~60.6 s	B

Road segments were chosen based on their consistent pavement quality, traffic flow, and significant elevation changes. Mounting Position and other test details are discussed in Section 2.5.

**Table 4:** Details of test numbers, air temperature, wind speed, wind direction, relative humidity at the start of the test, and engaged gear during the test.

Test Number	Air Temperature	Relative Humidity	Wind Speed	Wind Direction	Engaged Gear	Track Temperature
1	12 °C	61	3.1 m·s^−1^	South	4th and 5th	14 °C
2	12 °C	60	3.1 m·s^−1^	South	4th and 5th	14 °C
3	12 °C	61	3.9 m·s^−1^	South	4th and 5th	15 °C
4	11 °C	62	3.1 m·s^−1^	South	4th and 5th	12 °C
5	9 °C	76	1.9 m·s^−1^	South	4th and 5th	12 °C

**Table 5:** Details of the tire during the test.

Tire Brand	Tire Position	Tread Depth	Tire Pressure	Tire–Road Peak Friction Coefficient
SAVA 195/5 R15 Eskimo HP 85H 3PMSF	Front left	8.1 mm	210 kPa	0.9
	Front right	8.2 mm	210 kPa	0.9
	Rear left	8.3 mm	200 kPa	0.9
	Rear right	8.3 mm	200 kPa	0.9

**Table 6:** Reference values for different SI units according to ANSI S1.6 -2016 [39].

	Sound	Acceleration	Velocity	Force	Voltage
ref	$2\times 10^{-5}\;\mathrm{Pa}$	$1\times 10^{-6}\;m{\cdot s}^{-2}$	$1\times 10^{-6}\;\mathrm{mm}\cdot s^{-1}$	$1\times 10^{-6}\;N$	1 V

**Table 7:** Number of octave bands for N-point FFT [26].

N	512	1024	2048	4096	8192
1	6	7	8	9	10
1/3	18	21	24	27	30

**Table 8:** Comfort ratings and results obtained from six tests.

Vehicle Model	Test Drive Number	Track	Speed (km/h)	RMS Acceleration (a_w_) m·s^−2^	Comfort Rating ISO 2631-1	Extended Comfort Rating Based on a Custom Scale from ISO 2631-1	Extended Comfort Rating in Percentage
Opel Astra	1	A	30	0.812	Uncomfortable	Fairly uncomfortable to uncomfortable	67.67%
	2	B	50	0.539	Fairly uncomfortable	A little uncomfortable to fairly uncomfortable	85.82%
Skoda Octavia	3	A	30	0.668	Fairly uncomfortable	Fairly uncomfortable	74.31%
	4	B	50	0.432	A little uncomfortable	A little uncomfortable	83.04%
Volkswagen Passat	5	A	30	0.712	Fairly uncomfortable	Fairly uncomfortable	66.59%
	6	B	50	0.272	Not uncomfortable	Not uncomfortable	97.15%

**Table 9:** Comparison of the proposed Ride Comfort application with published baseline methods.

Vehicle Model	Test Drive Number	Proposed $a_{w}$	Zhao et al. $a_{w}$	Wawryszczuk et al. $a_{w}$	Δ Zhao (%)	Δ Wawryszczuk (%)
Opel Astra	1	0.812	0.978	0.978	−17	−17
	2	0.539	0.673	0.670	−19.9	−19.5
Skoda Octavia	3	0.668	0.706	0.705	−5.4	−5.2
	4	0.432	0.439	0.441	−1.6	−2
Volkswagen Passat	5	0.712	0.688	0.699	+3.4	+1.8
	6	0.272	0.234	0.231	+16.5	+18

**Figure A1:**
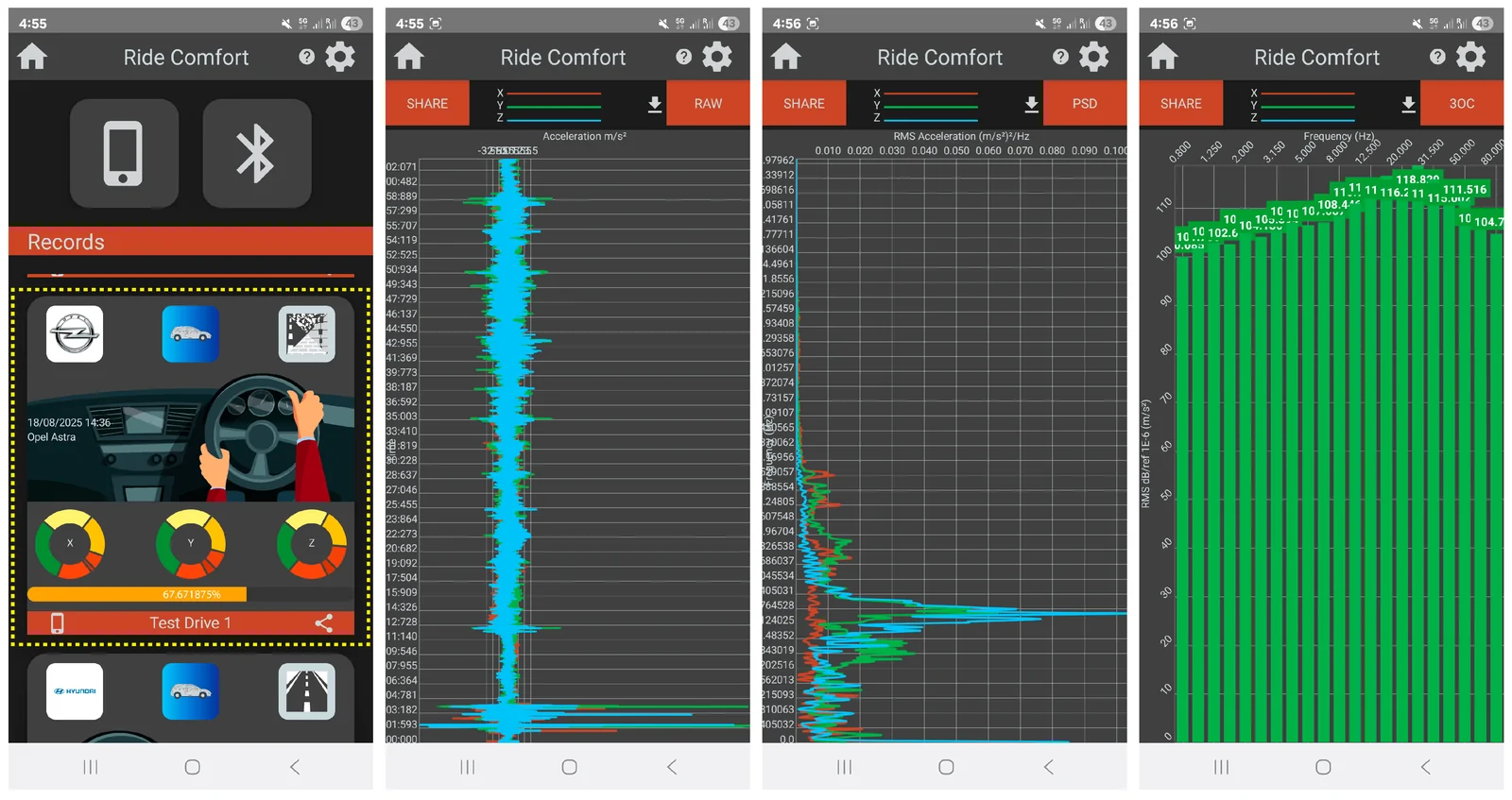
Test Drive 1 results (Opel Astra, Prague, 30 km/h).

**Figure A2:**
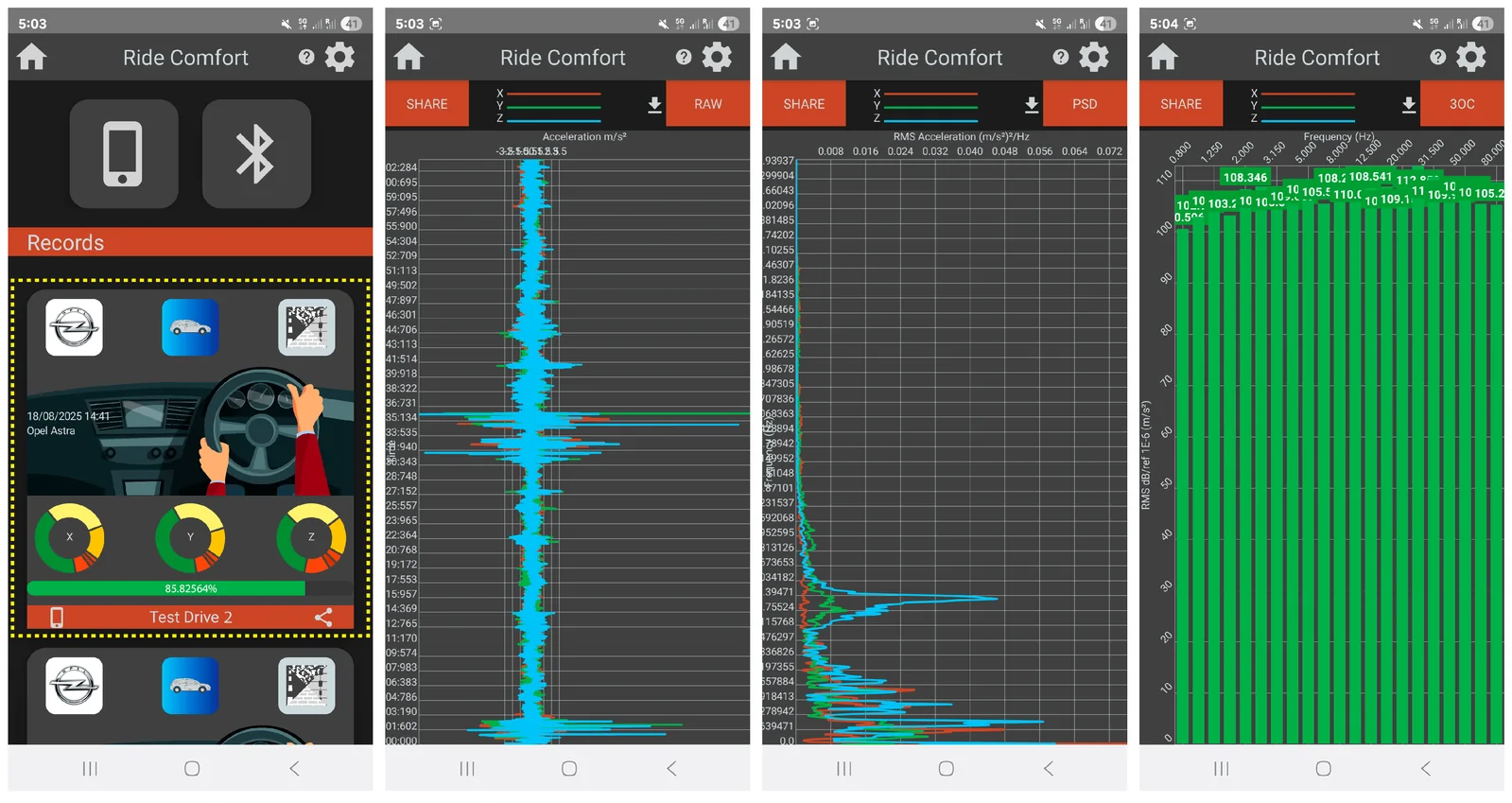
Test Drive 2 results (Opel Astra, Prague, 50 km/h).

**Figure A3:**
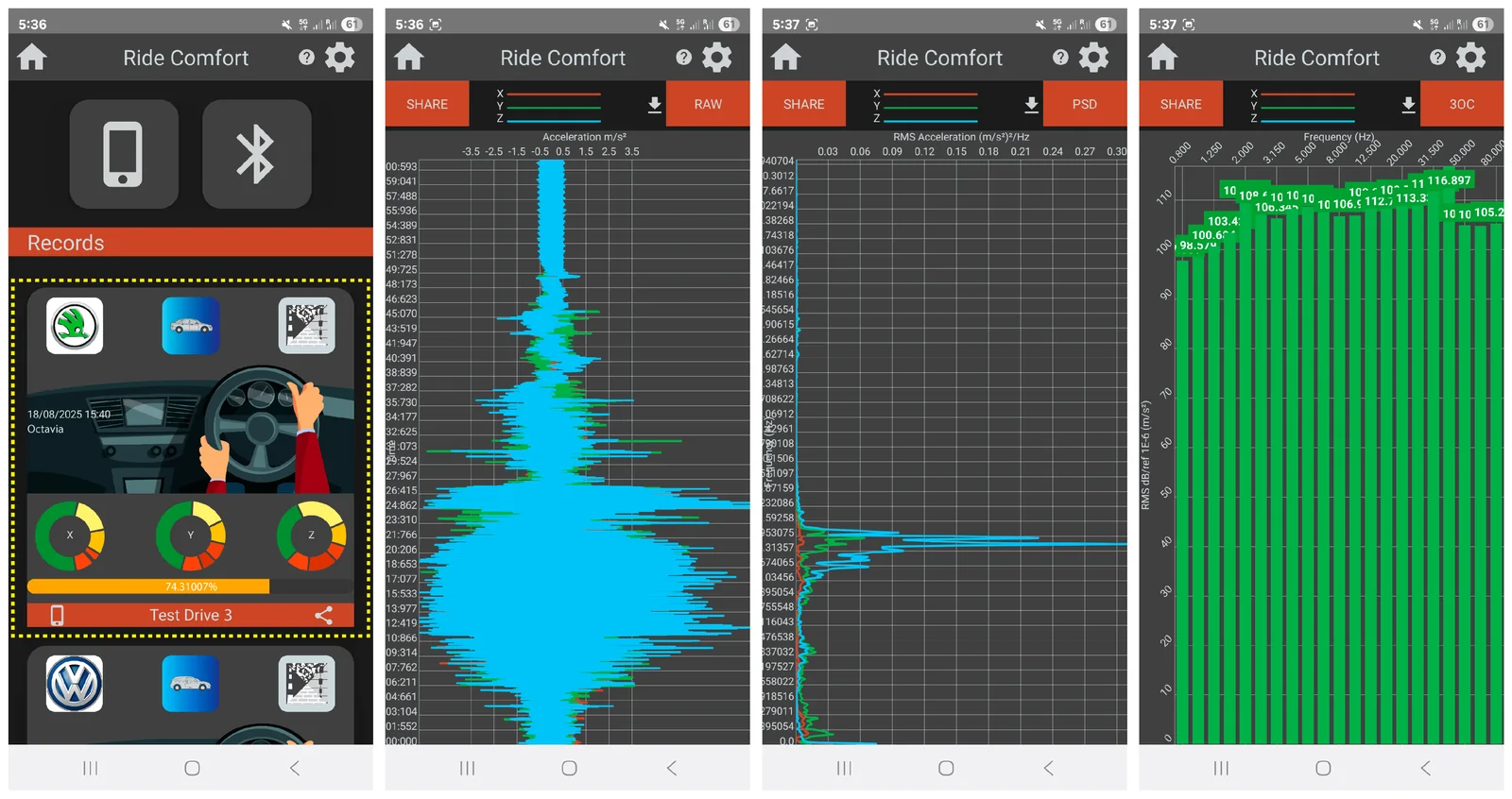
Test Drive 3 results (Skoda Octavia, Prague, 30 km/h).

**Figure A4:**
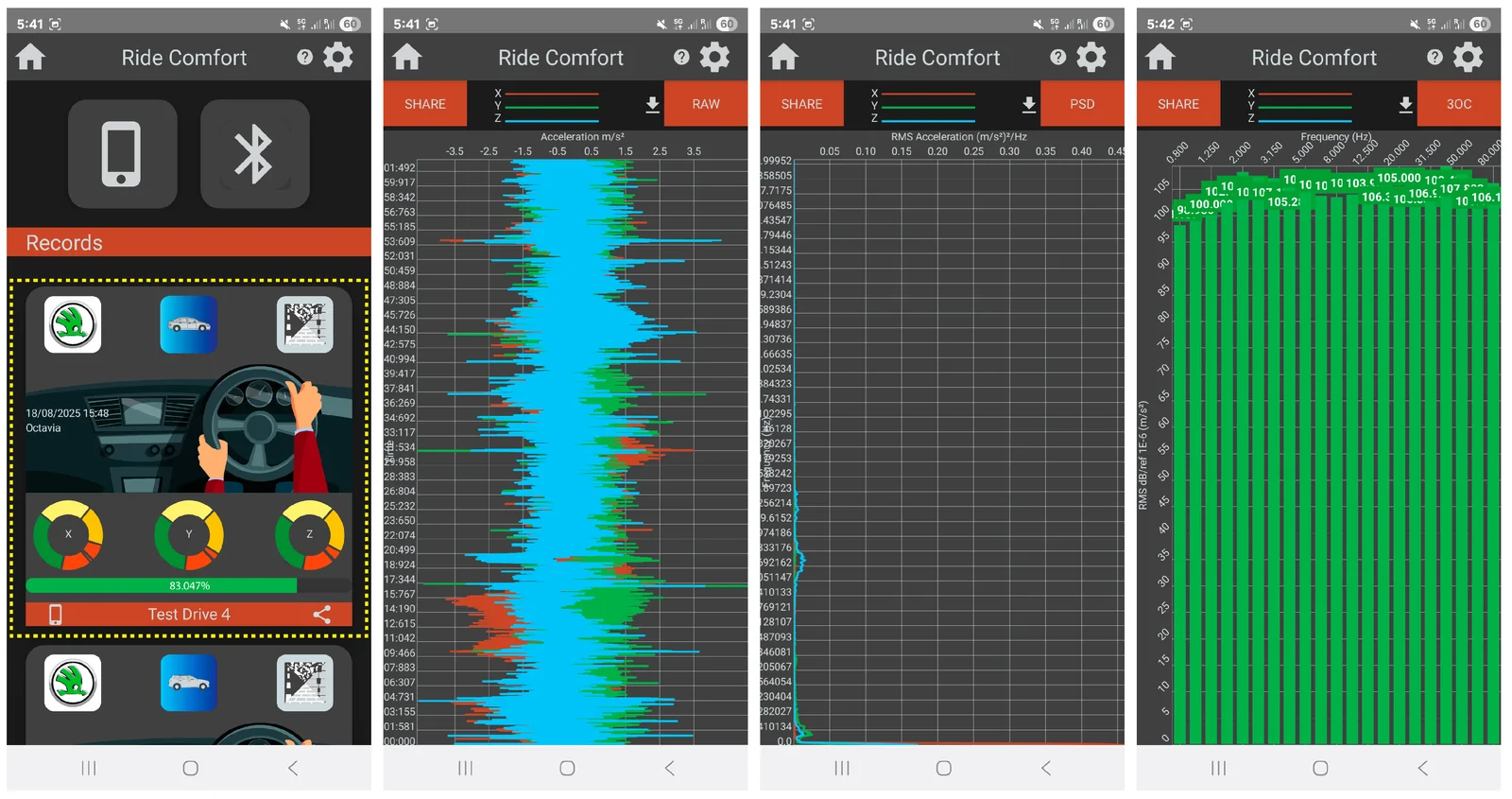
Test Drive 4 results (Skoda Octavia, Prague, 50 km/h).

**Figure A5:**
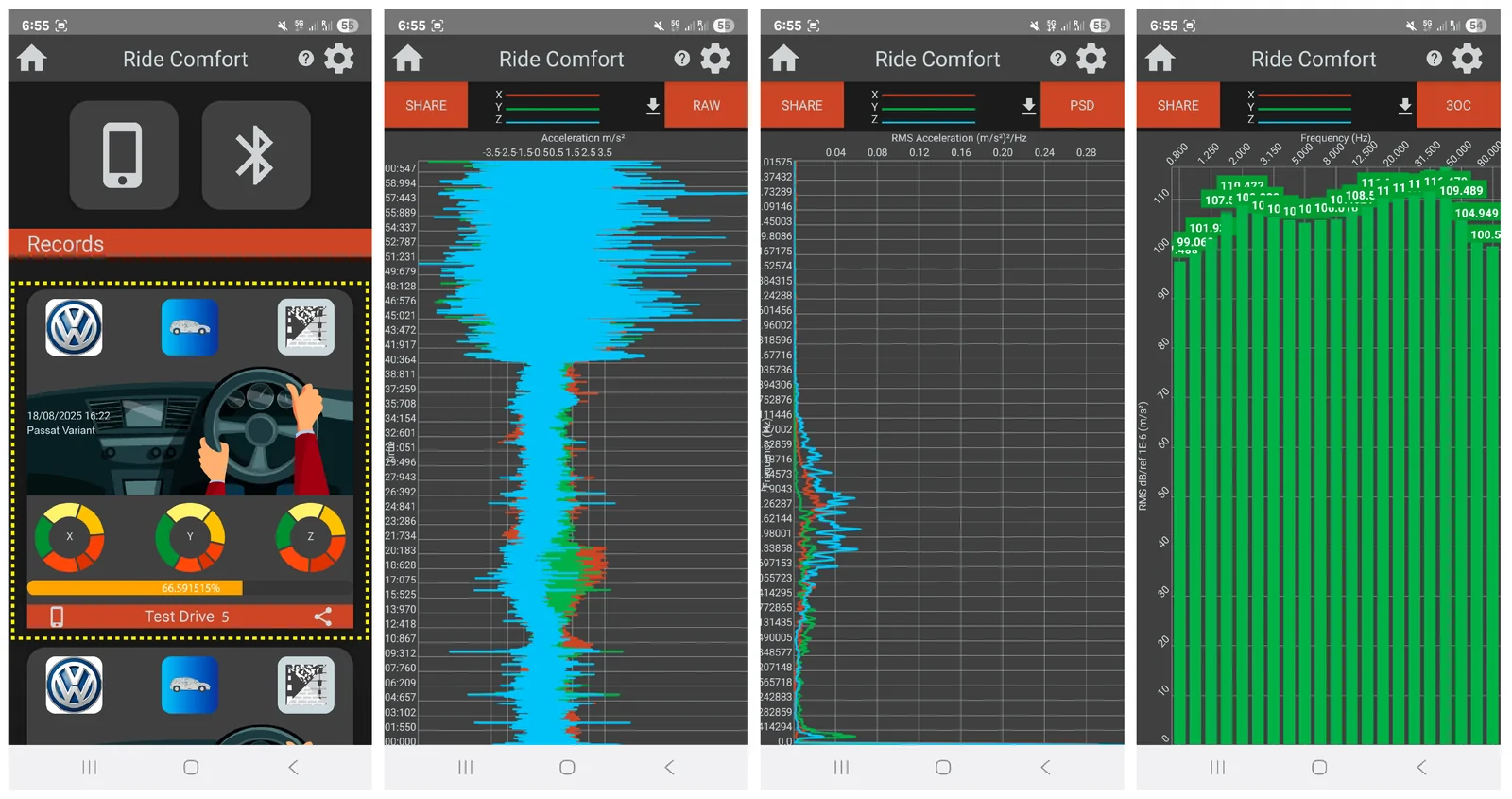
Test Drive 5 results (Volkswagen Passat, Prague, 30 km/h).

**Figure A6:**
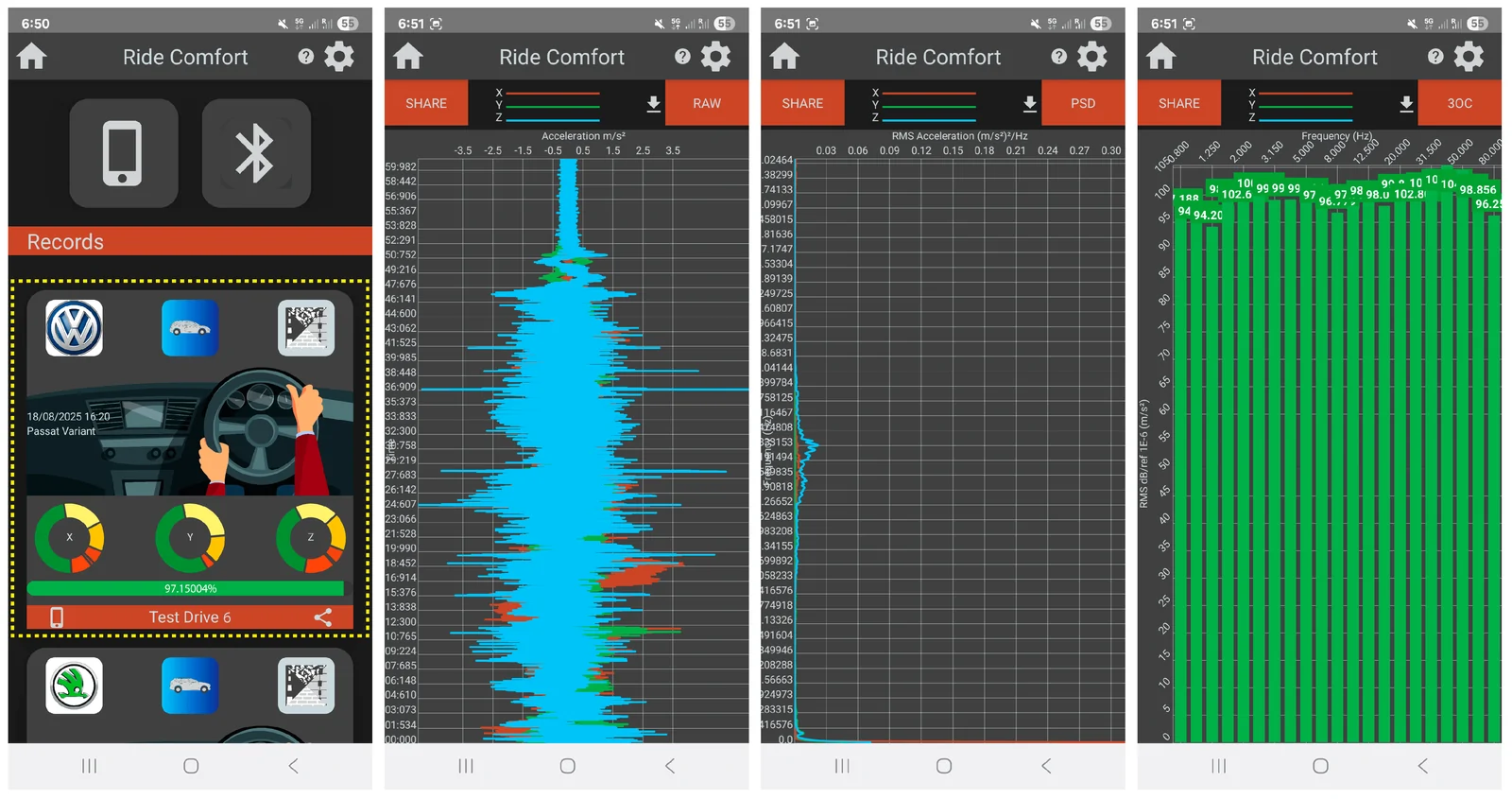
Test Drive 6 results (Volkswagen Passat, Prague, 50 km/h).

**Figure A7:**
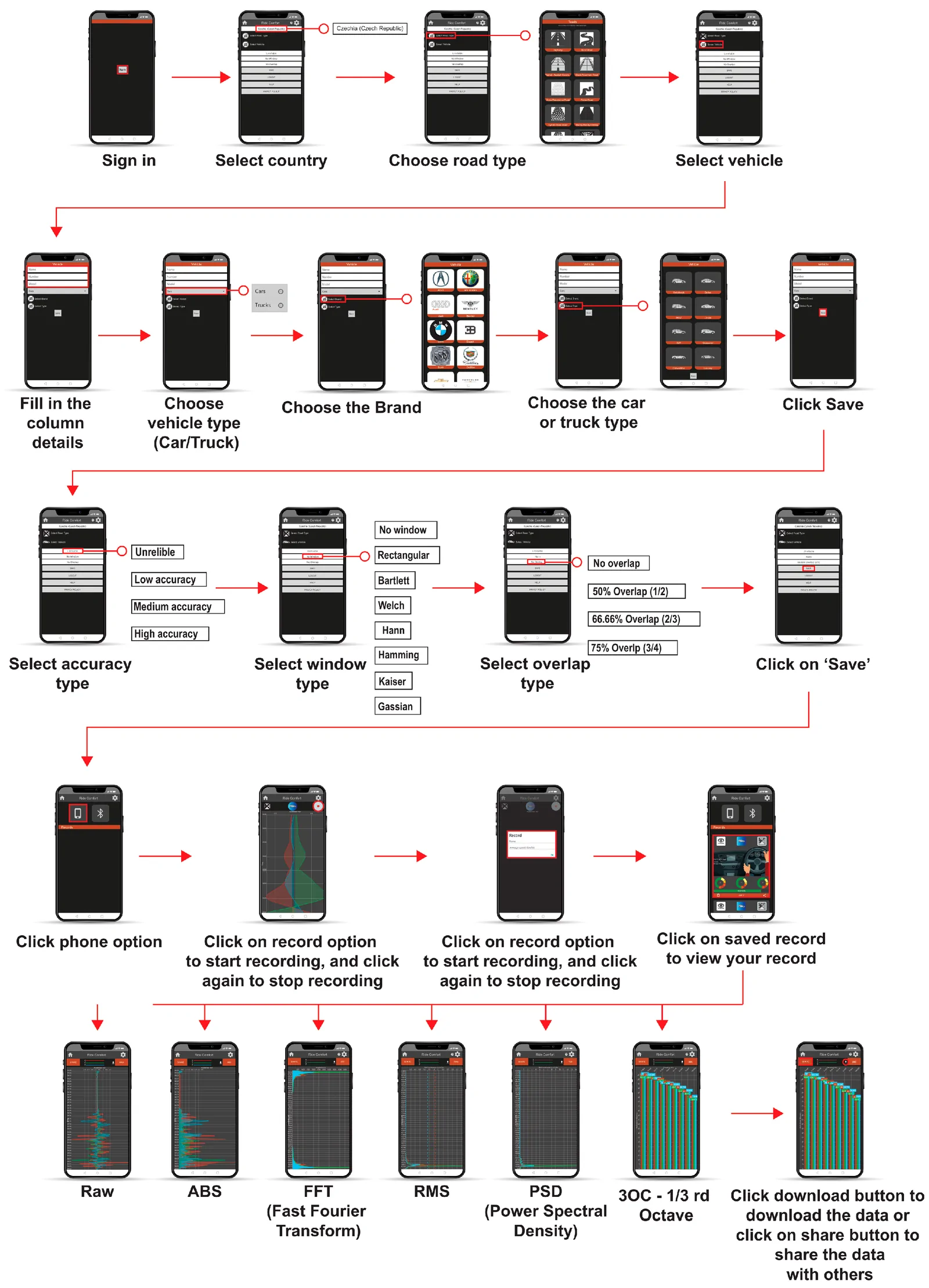
Illustrated workflow of the application, reproduced from the author M.N.R.’s MSc thesis [26].

## Data Availability

The raw vibration records, frequency-weighted RMS values, PSD results, and 1/3rd-octave spectra from all test drives, together with the Ride Comfort Android application (.xapk), are openly available in Zenodo at https://doi.org/10.5281/zenodo.16940237.
